# An organocatalytic route to 2-heteroarylmethylene decorated *N*-arylpyrroles

**DOI:** 10.3762/bjoc.9.168

**Published:** 2013-07-24

**Authors:** Alexandre Jean, Jérôme Blanchet, Jacques Rouden, Jacques Maddaluno, Michaël De Paolis

**Affiliations:** 1 Laboratoire de Chimie Moléculaire et Thio-organique, ENSICAEN, Université de Caen, CNRS; 2 Laboratoire des Fonctions Azotées et Oxygénées Complexes de l’IRCOF, CNRS UMR 6014 & FR 3038, Université et INSA de Rouen, Mont Saint-Aignan, France

**Keywords:** C–H oxidation, isomerization, *N*-arylpyrrole, organocatalysis

## Abstract

A concise and regioselective preparation of 2-heteroarylmethylene decorated *N*-arylpyrroles is described through a metal-free Mannich/Wittig/hydroamination sequence followed by isomerization of the *N*-arylpyrrolidine adducts. Furthermore, the C–H regioselective oxidation of these substrates is demonstrated, extending the molecular diversity and versatility of these scaffolds.

## Introduction

**Scheme 1:**
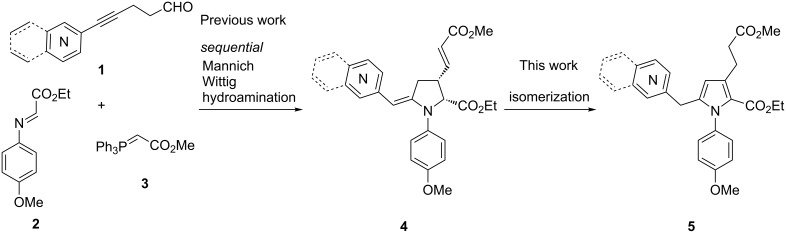
Synthetic approach toward *N*-arylpyrroles.

Due to their presence in some natural products [1] and pharmaceuticals [2–4], the preparation of *N*-arylpyrroles is an active field of investigation [5]. Depending on their substituents, *N*-arylpyrroles could also be electron donor/acceptor molecules with a dual fluorescence ability suggesting attractive optoelectronic applications [6–7]. If the *N*-arylation of pyrroles is possible by Ullmann-type condensation [8–10], the regioselective functionalization of pyrroles is less trivial when asymmetric substrates are targeted. An indirect solution, based on the construction of substituted pyrrolidines that oxidize into elaborated pyrroles, can be employed fruitfully [11–12]. We recently described a one-pot organo-catalyzed synthesis of *N*-heteroarylmethylene pyrrolidines **4** [13] from readily available aldehydes **1** and imine **2** by a sequence of Mannich coupling [14–24], Wittig olefination with phosphonium **3**, and proton-mediated hydroamination (Scheme 1). In the course of our investigations, we observed that pyrrolidine **4** could be converted into the corresponding pyrrole **5** by a simple isomerization, avoiding the use of oxidants. We describe herein the details of these observations and the scope of this methodology for the concise preparation of substituted 2-heteroaromatic decorated *N*-arylpyrroles.

## Results and Discussion

**Scheme 2:**
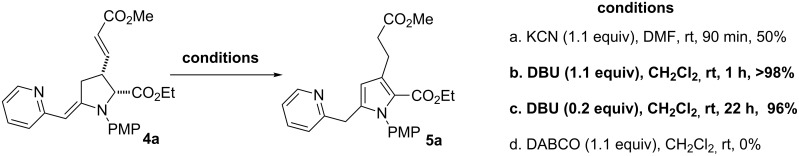
Isomerization of pyrrolidine **4a** (PMP: 4-methoxyphenyl).

We first observed the unexpected formation of pyrrole **5a** in 50% yield after treatment of pyrrolidine **4a** with KCN in DMF (Scheme 2, conditions a). Although obtained in modest yield, we found the original and unique structure of the substituted pyrrole **5a** interesting, especially with the 2*-*pyridylmethylene decoration. In an attempt to rationalize the formation of **5a**, we hypothesized that KCN acted as a nucleophilic and weak base since the level of oxidation of **4a** and **5a** was the same. To improve the efficiency of the transformation, a stronger nucleophilic base such as DBU (1,8-diazabicyclo[5.4.0]undec-7-ene) was tested [25]. Pleasingly, when pyrrolidine **4a** was exposed to DBU in CH_2_Cl_2_, **5a** was obtained in excellent yield (98%, 1 h, conditions b; Scheme 2). The reaction can also be promoted by a catalytic amount of DBU (0.2 equiv) delivering **5a** (96%) after prolonged reaction time (22 h, conditions c, Scheme 2). Interestingly and despite its strong nucleophilic character, DABCO (1,4-diazabicyclo[2.2.2]octane) was unable to promote the isomerization (conditions d, Scheme 2) and the starting material was recovered.

**Scheme 3:**
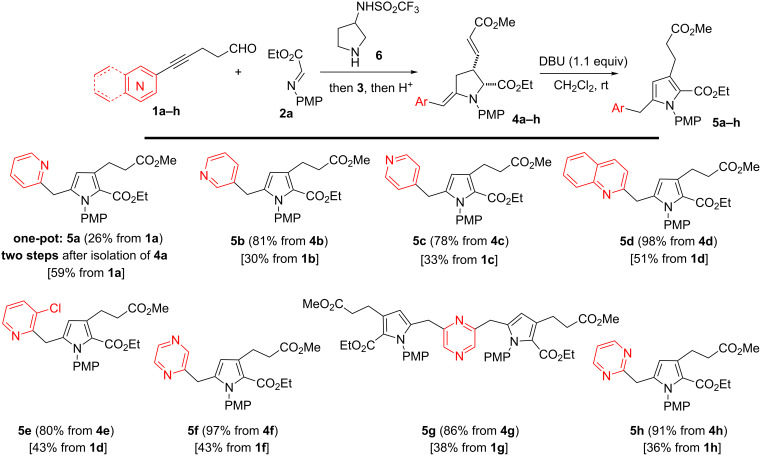
Preparation of *N*-arylpyrroles **5a–h** (unless otherwise specified, yields in brackets refer to the isomerization step while yields in square brackets refer to the two-step procedure from the corresponding aldehyde).

As presented in Scheme 3, the methodology was next attempted in a one-pot process. Hence, the transformation of aldehyde **1a** and imine **2a** into pyrrolidine **4a** was followed by the introduction of DBU leading to pyrrole **5a** in 26% yield. However, proceeding stepwise and isolating the pyrrolidine **4a** by a simple filtration on silica gel before isomerization is more rewarding: following this route, the global yield for the whole process reaches 59% yield. Applying this procedure, various 2-heteroarylmethylenepyrrolidines **4b–h** prepared from aldehydes **1b–h** and imine **2a** were exposed to DBU (1.1 equiv). Pleasingly, pyrrolidines **4b–h** were transformed into the corresponding pyrroles **5b–i** with homogeneous efficiency. Hence, the chemistry proved to be compatible with substrates containing *meta*-, *para*-pyridyl and quinolinyl substituents, allowing the preparation of **5b** (81%), **5c** (78%) and **5d** (98%). Pyrrolidine **4e** containing an electronically deficient pyridyl residue was also converted into **5e** (80%) while pyrrolidine **4f** bearing a pyrazine core underwent aromatization with high efficiency to give **5f** (97%). The *C*_2_-symmetric scaffold **4g** was efficiently converted into **5g** (86%) and similar treatment of pyrimidine **4h** provided pyrrole **5h** in high yield (91%).

**Scheme 4:**
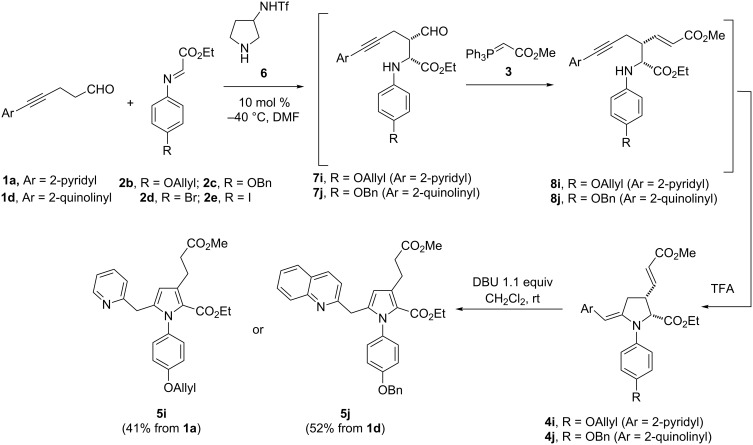
Variation of imines.

The Mannich coupling was next attempted with different imines **2b–e** in order to modulate the nature of the aryl moiety (Scheme 4). The electronic nature of the aniline being crucial for the stability of the imine and the hydroamination step, electronically rich anilines were selected to form imines **2b**,**c**. Hence, when imines **2b**,**c** were exposed to aldehyde **1a** in the presence of catalyst **6** (available in racemic form), the Mannich adducts **7i**,**j** were obtained and directly reacted with phosphonium salt **3**. In line with our procedure, the resulting acyclic anilines **8i**,**j** were then exposed to TFA to promote the cyclization into pyrrolidines **4i**,**j** which upon treatment with DBU were converted into pyrroles **5i**,**j** in 41% and 52% overall yields. While *p*-alkoxy substituted (R = OAllyl, OBn) anilines are compatible, the methodology proved troublesome with *o*-alkoxy substituted anilines, the main limitation being the formation of the corresponding imines. Similarly, imine **2d** prepared from *para*-bromoaniline was found to be unstable and only degradation was observed during the Mannich reaction. When imine **2e**, derived from the *para*-iodoaniline, was engaged in the process, the hydroamination step turned out to be problematic, which prevented the isolation of the corresponding pyrrolidine.

**Scheme 5:**
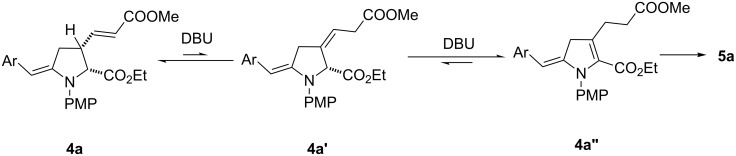
Possible mechanism.

Even if not completely elucidated, a mechanism of the isomerization can be suggested in which the acrylate moiety is crucial. Indeed, without this unsaturation, it was not possible to observe the isomerization of the *exo*-enamine into the *endo* compound under basic treatment [26]. These observations suggest that DBU or KCN behave as base to promote the deconjugation of the acrylate moiety of **4a** [27]. The resulting product **4a’** would lead under basic treatment to pyrroline **4a”** from which aromatization to **5a** would be expected to follow (Scheme 5).

**Scheme 6:**
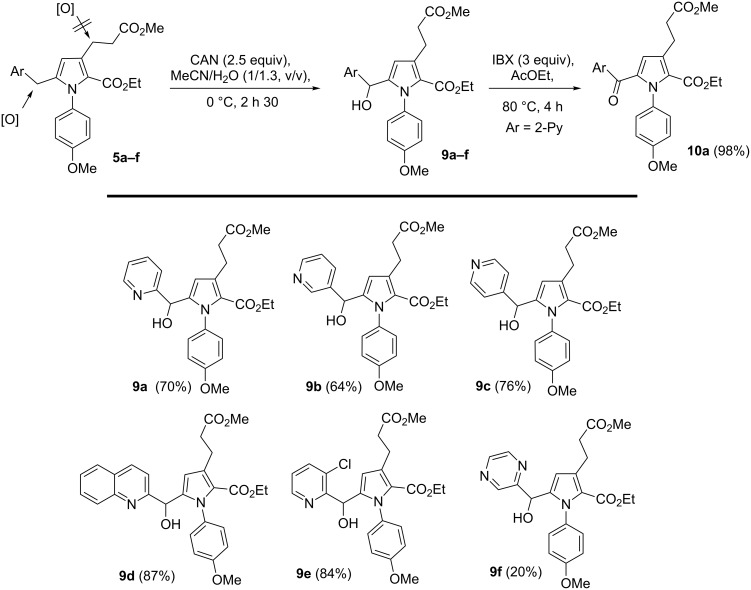
Bis(heteroaryl)methylene oxidation of **5a–f**.

Having established a practical methodology for the preparation of substituted *N*-arylpyrroles, we next undertook synthetic transformations to extend the molecular diversity of the substrates. While attempts to perform an oxidation of the bis(heteroaryl)methylene position with elemental sulfur [28] or SeO_2_ failed, the oxidation of this methylene position was regioselectively carried out by treatment of **5a–e** with (NH_4_)_2_Ce(NO_3_)_6_ (CAN), delivering the alcohols **9a–e** (Scheme 6). The methylene oxidation was especially efficient with substrates containing mononitrogenated heteroaryl substituents, with yields ranging from 64–87%. Oxidation under the same conditions was found to be more troublesome with pyrazine **5f** since alcohol **9f** was isolated in only 20% yield. Similar treatment of pyrazine **5g** and pyrimidine **5h** gave a complex mixture of products. While the oxidation of the bis(aryl)methylene position with CAN has been reported [29], this is the first example of bis(heteroaryl)methylene oxidation employing this reagent [30]. In order to increase the local electron deficiency of the scaffold, **9a** was oxidized with 2-iodoxybenzoic acid (IBX) into ketone **10a** (98%), which presents an ideal push–pull configuration tunable with the pH by protonation of the pyridine ring. This is likely to lead to applications of **10a** such as for new water-soluble molecular probes.

## Conclusion

A new catalytic and regioselective preparation of substituted *N*-arylpyrroles decorated with various 2-heteroaromatic scaffolds is reported. Based on the isomerization of pyrrolidines prepared by a simple and efficient sequence of Mannich/Wittig olefination/hydroamination reactions, no oxidant or metallic salts were employed [31]. This study also led us to investigate the feasibility of this process with different anilines and enlarge the molecular diversity of the scaffold. So far the methodology is limited to electron-rich anilines due to the formation and reactivity of the corresponding imines and the stability of the Mannich adduct for the hydroamination step. However, this electronic configuration is ideal for the preparation of electron donor/acceptor *N*-arylpyrroles as demonstrated in this study. In addition, we documented an efficient C–H oxidation of the bis(heteroaryl)methylene position promoted by CAN.

## Experimental

**General:**^ 1^H and ^13^C NMR spectra were recorded in deuterated chloroform on Bruker Avance DPX 400 or 300 spectrometers and were referenced to residual chloroform (7.26 ppm, ^1^H; 77.00 ppm, ^13^C). Chemical shifts are expressed in parts per million (ppm). Data for ^1^H are reported as follows: chemical shift (δ ppm), multiplicity (s = singlet, d = doublet, t = triplet, q = quartet, quint = quintuplet, sept = septuplet, m = multiplet), coupling constant (Hz), integration. Mass spectra and high-resolution mass spectra (HRMS) were obtained on a Waters-Micromass Q-Tof micro instrument. IR data were obtained on a PerkinElmer Spectrum 100 FTIR-spectrometer with only major peaks being reported. Thin-layer chromatography (TLC) was performed on silica gel 60 F_254_ plates (0.1 mm, Merck). Visualization was accomplished with UV (254 nm) or KMnO_4_ staining solutions. Chromatographic separations were achieved on silica-gel columns (Kieselgel 60, 40–63 μm, Merck).

Technical grade *N*,*N*-dimethylformamide and dichloromethane were used for this work. Following our procedure [13], catalyst **6** was prepared from (±)-1-benzyl-3-aminopyrrolidine [18471-40-4]. *N*-Arylimino ethyl glyoxylates **2a–c** were prepared by a condensation of ethyl glyoxylate and arylamines in toluene (*c* = 1 M) with MgSO_4_ at room temperature. IBX (2-iodoxybenzoic acid) was prepared according to standard procedures.

**Ethyl 3-(3-methoxy-3-oxopropyl)-1-(4-methoxyphenyl)-5-(pyridin-2-ylmethyl)-1*H*-pyrrole-2-carboxylate (5a):** Representative procedure: In a flask containing a stirred solution of **4a** (432 mg, 1.02 mmol, 1.0 equiv) in CH_2_Cl_2_ (10.2 mL) at room temperature was introduced DBU (168 μL, 1.12 mmol). The mixture was allowed to react at this temperature for 1 h. Then, the volatiles were removed under reduced pressure and the crude was purified by flash column chromatography (CH_2_Cl_2_/MeOH 99:1) on silica gel to yield **5a** (432 mg, 99%) as an orange oil. ^1^H NMR (300 MHz, CDCl_3_) 1.08 (t, *J* = 7.1 Hz, 3H, OCH_2_*CH_3_*), 2.65 (br t, *J* = 7.9 Hz, 2H), 3.11 (br t, *J* = 7.9 Hz, 2H), 3.67 (s, 3H), 3.81 (s, 3H), 3.83 (s, 2H), 4.06 (q, *J* = 7.1 Hz, 2H), 5.92 (s, 1H), 6.83 (d, *J* = 8.8 Hz, 2H), 6.86 (d, *J* = 7.8 Hz, 1H), 6.98 (d, *J* = 8.8 Hz, 2H), 7.06 (m, 1H), 7.52 (td, *J* = 1.5, 7.8 Hz, 1H), 8.45 (d, *J* = 4.3 Hz, 1H) ppm*; *^13^C NMR (75 MHz, CDCl_3_) 173.7, 160.8, 158.9, 158.4, 149.1, 137.6, 136.3, 132.4, 132.1, 128.8 (2*CH), 122.8, 121.3, 120.7, 113.5 (2*CH), 110.4, 59.3, 55.2, 51.3, 35.8, 34.9, 23.5, 13.9 ppm*;* IR: 2920, 1690, 1512, 1438, 1245, 1169, 1080, 910, 727 cm^−1^; HRMS (ESI^+^): (M + H)^+^ calcd for C_24_H_27_N_2_O_5_, 423.1920; found, 423.1926; *R*_f_ 0.15 (CH_2_Cl_2_/MeOH 99:1).

**Ethyl 5-(hydroxy(pyridin-2-yl)methyl)-3-(3-methoxy-3-oxopropyl)-1-(4-methoxyphenyl)-1*H*-pyrrole-2-carboxylate (9a):** Representative procedure: In a flask containing a well stirred solution of CAN (269 mg, 0.490 mmol, 3.0 equiv) in H_2_O (3.1 mL) at 0 °C was introduced dropwise a solution of **5a** (69 mg, 0.164 mmol in 2.4 mL of CH_3_CN) over 10 min. Then, the mixture was allowed to react at this temperature for 3 h and was quenched by the addition of an aqueous solution of Na_2_S_2_O_3_ (1 M). The resulting mixture was extracted with AcOEt (3×), and the combined organic layers were washed with brine, dried over MgSO_4_, and filtered. The volatiles were removed under reduced pressure to give 58 mg of the crude product, which was purified by flash column chromatography (CH_2_Cl_2_/MeOH 99:1) on silica gel to yield **9a** (49 mg, 71%) as an orange oil. ^1^H NMR (300 MHz, CDCl_3_) 1.08 (t, *J* = 7.1 Hz, 3H, OCH_2_*CH_3_*), 2.60 (br t, *J* = 7.9 Hz, 2H), 3.06 (m, 2H), 3.64 (s, 3H), 3.82 (s, 3H), 4.06 (q, *J* = 7.1 Hz, 2H), 5.43 (s, 1H), 5.85 (s, 1H), 6.87 (ddd, *J* = 2.8, 8.6, 11.4 Hz, 2H), 7.05 (d, *J* = 7.8 Hz, 1H), 7.08–7.22 (m, 3H), 7.61 (dt, *J* = 1.6, 7.8 Hz, 1H), 8.45 (d, *J* = 4.8 Hz, 1H) ppm; ^13^C NMR (75 MHz, CDCl_3_) 173.7 (Cq), 160.9 (Cq), 159.1 (Cq), 158.9 (Cq), 147.7 (CH), 141.0 (Cq), 136.6 (CH), 132.0 (Cq), 131.7 (Cq), 129.4 (2*CH), 122.6 (CH), 121.7 (Cq), 121.3 (CH), 113.6 (CH), 113.4 (CH), 110.1 (CH), 67.1 (CH), 59.6 (CH_2_), 55.4 (CH_3_), 51.4 (CH_3_), 35.0 (CH_2_), 23.5 (CH_2_), 13.9 (CH_3_) ppm; IR: 3375, 2927, 1733, 1688, 1512, 1436, 1368, 1295, 1081, 1030, 999, 834, 753 cm^−1^; HRMS (ESI^+^): (M + H)^+^ calcd for C_24_H_27_N_2_O_6_, 439.1869; found, 439.1886; *R*_f_ 0.1 (CH_2_Cl_2_/MeOH 9:1).

**Ethyl 3-(3-methoxy-3-oxopropyl)-1-(4-methoxyphenyl)-5-picolinoyl-1*H*-pyrrole-2-carboxylate (10a):** A solution of **9a** (26 mg, 0.0593 mmol) in AcOEt (0.6 mL) was treated with IBX (50 mg, 0.178 mmol, 3 equiv). The suspension was stirred at 80 °C for 4 h before being brought to rt and filtered. Evaporation of the volatile led to analytically pure **10a** which can be further purified by flash chromatography (CH_2_Cl_2_/MeOH 99.3:0.7) to yield 24 mg of **10a**. ^1^H NMR (400 MHz, CDCl_3_) 1.10 (t, *J* = 7.1 Hz, 3H), 2.69 (br t, *J* = 7.8 Hz, 2H), 3.14 (br t, *J* = 7.8 Hz, 2H), 3.67 (s, 3H), 3.82 (s, 3H), 4.11 (q, *J* = 7.1 Hz, 2H), 6.90 (d, *J* = 8.8 Hz, 2H), 7.19 (d, *J* = 8.8 Hz, 2H), 7.21 (s, 1H), 7.41–7.46 (ddd, *J* = 1.3, 4.7, 6.1 Hz, 1H), 7.80 (dt, *J* = 1.6, 7.7 Hz, 1H), 7.86 (br d, *J* = 7.7 Hz, 1H), 8.68 (d, *J* = 4.7 Hz, 1H) ppm; ^13^C NMR (100 MHz, CDCl_3_) 182.9 (Cq), 173.5 (Cq), 160.7 (Cq), 159.1 (Cq), 155.4 (Cq), 148.3 (CH), 136.9 (CH), 133.3 (Cq), 133.0 (Cq), 130.6 (Cq), 128.5 (2*CH), 127.8 (Cq), 126.2 (CH), 124.0 (CH), 123.0 (CH), 113.3 (2*CH), 60.5 (CH_2_), 55.3 (CH_3_), 51.5 (CH_3_), 34.9 (CH_2_), 23.2 (CH_2_), 13.8 (CH_3_) ppm; IR: 2952, 1716, 1647, 1511, 1437, 1335, 1230, 1164, 1085, 831, 746, 689 cm^−1^; HRMS (ESI^+^): (M + H)^+^ calcd for C_24_H_25_N_2_O_6_; 437.1713; found, 437.1700; *R*_f_ 0.25 (CH_2_Cl_2_/MeOH 99:1).

## Supporting Information

File 1 Physical and spectroscopic data of **5b–j**, **9b–e** and ^1^H and ^13^C spectra of all new compounds.
