# Supplementary material for: An organocatalytic route to 2-heteroarylmethylene decorated N-arylpyrroles
Source: Beilstein J Org Chem. 2013 Jul 24;9:1480–6. doi: 10.3762/bjoc.9.168 (PMC3740684; doi:10.3762/bjoc.9.168)
Supplement: File 1 — Physical and spectroscopic data of 5b–j, 9b–e and 1H and 13C spectra of all new compounds. [file Beilstein_J_Org_Chem-09-1480-s001.pdf]

Supporting Information

for

An organocatalytic route to 2-heteroarylmethylene decorated *N*-arylpyrroles

Alexandre Jean[1,2], Jérôme Blanchet[*,1], Jacques Rouden[1], Jacques Maddaluno[2] and Michaël De Paolis[*,2]

Address: [1]Laboratoire de Chimie Moléculaire et Thio-organique, ENSICAEN, Université de Caen, CNRS, 6 boulevard du Marechal Juin, 14050 Caen, France and [2]Laboratoire des Fonctions Azotées et Oxygénées Complexes de l'IRCOF, CNRS UMR 6014 & FR 3038, Université et INSA de Rouen, Mont Saint-Aignan, France

Email: Jérôme Blanchet - jerome.blanchet@ensicaen.fr ; Michaël De Paolis - michael.depaolis@univ-rouen.fr

*Corresponding author

Physical and spectroscopic data of 5b–j, 9b–e and ^{1}H and ^{13}C spectra of all new compounds

Ethyl 3-(3-methoxy-3-oxopropyl)-1-(4-methoxyphenyl)-5-(pyridin-3-ylmethyl)-1*H*-pyrrole-2-carboxylate (5b)

^{1}H NMR (300 MHz, $CDCl_3$) δ = 1.08 (t, 3H, *J* = 7.1 Hz, OCH_2CH_3), 2.63 (br t, 2H, *J* = 7.7 Hz), 3.11 (br t, 2H, *J* = 7.7 Hz), 3.63 (s, 2H), 3.67 (s, 3H), 3.82 (s, 3H), 4.06 (q, 2H, *J* = 7.1 Hz), 5.90 (s, 1H), 6.85 (d, 2H, *J* = 8.9 Hz), 6.96 (d, 2H, *J* = 8.9 Hz), 7.13 (m, 1H), 7.27 (m, 1H), 8.15 (br s, 1H), 8.41 (d, 1H, *J* = 4.3 Hz) ppm; ^{13}C NMR (75 MHz, $CDCl_3$) δ = 173.7, 160.8, 159.2, 149.9, 147.7, 137.8, 136.0, 134.1, 132.4, 132.0, 128.9 (2*CH), 123.2, 121.1, 113.7 (2*CH), 110.4, 59.5, 55.4, 51.4, 35.1, 30.5, 23.5, 14.0 ppm; IR 2927, 1733, 1690, 1512, 1447, 1368, 1246, 1167, 1079, 1027, 833, 799, 713 cm^{-1}; HRMS (ESI^+) calcd for $(M + H)^+$ $C_{24}H_{27}N_2O_5$: 423.1920, found: 423.1910; R_f = 0.15 (CH_2Cl_2/MeOH: 99/1).

Ethyl 3-(3-methoxy-3-oxopropyl)-1-(4-methoxyphenyl)-5-(pyridin-4-ylmethyl)-1*H*-pyrrole-2-carboxylate (5c)

^{1}H NMR (300 MHz, $CDCl_3$) δ = 1.09 (t, 3H, *J* = 7.1 Hz, OCH_2CH_3), 2.65 (br t, 2H, *J* = 7.7 Hz), 3.13 (br t, 2H, *J* = 7.7 Hz), 3.64 (s, 2H), 3.68 (s, 3H), 3.81 (s, 3H), 4.06 (q, 2H, *J* = 7.1 Hz), 5.96 (s, 1H), 6.77–6.96 (m, 6H), 8.41 (br d, 2H, *J* = 4.4 Hz) ppm; ^{13}C NMR (75 MHz, $CDCl_3$) δ = 173.7, 160.8, 159.2, 149.6 (2*CH), 147.8, 136.8, 132.4, 132.0, 128.9 (2*CH), 123.8, 121.3, 113.7 (2*CH), 110.7, 59.5, 55.4, 51.5, 35.1, 32.5, 23.5, 14.0 ppm; IR 2927, 1734, 1695, 1596, 1512, 1444, 1295, 1246, 1153, 1079, 1032, 833 cm^{-1}; HRMS (ESI^+) calcd for $(M + H)^+$ $C_{24}H_{27}N_2O_5$: 423.1920, found: 423.1905; R_f = 0.25 (CH_2Cl_2/MeOH: 99/1).

Ethyl 3-(3-methoxy-3-oxopropyl)-1-(4-methoxyphenyl)-5-(quinolin-2-ylmethyl)-1*H*-pyrrole-2-carboxylate (5d)

^{1}H NMR (300 MHz, $CDCl_3$) δ = 1.09 (t, 3H, *J* = 7.1 Hz, OCH_2CH_3), 2.63 (br t, 2H, *J* = 7.9 Hz), 3.11 (br t, 2H, *J* = 7.9 Hz), 3.64 (s, 3H), 3.77 (s, 3H), 4.07 (q + s, 2H + 2H, *J* = 7.1 Hz), 5.93 (s, 1H), 6.81 (d, 2H, *J* = 8.9 Hz), 7.02 (d, 2H, *J* = 8.9 Hz), 7.11 (d, 1H, *J* = 8.5 Hz), 7.51 (m, 1H), 7.68 (m, 1H), 7.77 (br d, 1H, *J* = 8.1 Hz), 7.98 (d, 1H, *J* = 8.5 Hz), 8.03 (d, 1H, *J* = 8.5 Hz) ppm; ^{13}C NMR (75 MHz, $CDCl_3$) δ =173.7 (Cq), 160.9 (Cq), 159.0 (Cq), 158.6 (Cq), 147.5 (Cq), 137.4 (Cq), 136.5 (CH), 132.5 (Cq), 132.1 (Cq), 129.5 (CH), 129.0 (2*CH), 128.8 (CH), 127.4 (CH), 126.7 (Cq), 126.1 (CH), 121.1 (CH), 120.9 (Cq), 113.7 (2*CH), 110.6 (CH), 59.4 (CH_2), 55.3 (CH_3), 51.4 (CH_3), 36.7 (CH_2), 35.0 (CH_2), 23.5 (CH_2), 14.0 (CH_3) ppm; IR 2920, 1733.8, 1688, 1600, 1512, 1245, 1168, 1079, 1033, 832 cm^{-1}; HRMS (ESI^+) calcd for $(M + H)^+$ $C_{28}H_{29}N_2O_5$: 473.2076, found: 473.2093; R_f = 0.2 (CH_2Cl_2/MeOH: 99/1).

Ethyl 5-((3-chloropyridin-2-yl)methyl)-3-(3-methoxy-3-oxopropyl)-1-(4-methoxyphenyl)-1*H*-pyrrole-2-carboxylate (5e)

^{1}H NMR (300 MHz, $CDCl_3$) δ = 1.10 (t, 3H, *J* = 7.1 Hz, OCH_2CH_3), 2.61 (br t, 2H, *J* = 7.9 Hz), 3.08 (br t, 2H, *J* = 7.9 Hz), 3.65 (s, 3H), 3.83 (s, 3H), 3.94 (s, 2H), 4.07 (q, 2H, *J* = 7.1 Hz), 5.70 (s, 1H), 6.87 (d, 2H, *J* = 8.9 Hz), 7.11 (d, 2H, *J* = 8.9 Hz), 7.12 (m, 1H), 7.59 (dd, 1H, *J* = 1.5, 8.1 Hz), 8.42 (dd, 1H, *J* = 1.5, 4.7 Hz) ppm; ^{13}C NMR (75 MHz, $CDCl_3$) δ = 173.8 (Cq), 160.8 (Cq), 159.0 (Cq), 155.5 (Cq), 147.2 (CH, 136.9 (CH), 136.7 (Cq), 132.4 (Cq), 132.2 (Cq), 131.2 (Cq), 128.8 (2*CH), 122.8 (CH), 120.6 (Cq), 113.6 (2*CH), 109.9 (CH), 59.3 (CH_2), 55.3 (CH_3), 51.4 (CH_3), 35.0 (CH_2), 33.4 (CH_2), 23.7 (CH_2), 13.9 (CH_3) ppm; IR 2952, 1734, 1695, 1512, 1444, 1370, 1296, 1246, 1173, 1081, 1035, 834 cm^{-1}; HRMS (ESI^+) calcd for $(M + H)^+$

$C_{24}H_{26}{}^{35}ClN_2O_5$: 457.1530, found: 457.1524; R_f = 0.2 (CH_2Cl_2/MeOH: 99/1). NB: py-CH_2 –pyrrol integrate for 1.4H on ^{1}H spectrum. No deuterium exchange was observed in CD_3OD/D_2O.

Ethyl 3-(3-methoxy-3-oxopropyl)-1-(4-methoxyphenyl)-5-(pyrazin-2-ylmethyl)-1*H*-pyrrole-2-carboxylate (5f)

^{1}H NMR (300 MHz, $CDCl_3$) δ = 1.09 (t, 3H, *J* = 7.1 Hz, OCH_2CH_3), 2.64 (br t, 2H, *J* = 7.9 Hz), 3.11 (br t, 2H, *J* = 7.9 Hz), 3.67 (s, 3H), 3.81 (s, 3H), 3.87 (s, 2H), 4.06 (q, 2H, *J* = 7.1 Hz), 5.91 (s, 1H), 6.84 (d, 2H, *J* = 8.9 Hz), 6.98 (d, 2H, *J* = 8.9 Hz), 8.10 (br s, 1H), 8.37 (br s, 1H), 8.42 (br s, 1H) ppm; ^{13}C NMR (75 MHz, $CDCl_3$) δ = 173.7 (Cq), 160.8 (Cq), 159.2 (Cq), 154.3 (Cq), 144.5 (CH), 144.0 (CH), 142.4 (CH), 136.1 (Cq), 132.5 (Cq), 132.0 (Cq), 128.9 (2*CH), 121.1 (Cq), 113.7 (2*CH), 110.8 (CH), 59.5 (CH_2), 55.4 (CH_3), 51.5 (CH_3), 35.0 (CH_2), 33.3 (CH_2), 23.5 (CH_2), 14.0 (CH_3) ppm; IR 2939, 1733, 1695, 1512, 1444, 1371, 1246, 1159, 1080, 1018, 834 cm^{-1}; HRMS (ESI^+) calcd for $(M + H)^+$ $C_{23}H_{26}N_3O_5$: 424.1872, found: 424.1856; R_f = 0.15 (CH_2Cl_2/MeOH: 99/1).

Diethyl 5,5'-(pyrazine-2,6-diylbis(methylene))bis(3-(3-methoxy-3-oxopropyl)-1-(4-methoxyphenyl)-1H-pyrrole-2-carboxylate) (5g)

^{1}H NMR (300 MHz, $CDCl_3$) δ = 1.09 (t, 6H, *J* = 7.1 Hz, OCH_2CH_3), 2.63 (br t, 4H, *J* = 7.8 Hz), 3.10 (br t, 4H, *J* = 7.8 Hz), 3.67 (s, 6H), 3.77 (s, 4H), 3.80 (s, 6H), 4.06 (q, 4H, *J* = 7.1 Hz), 5.90 (s, 2H), 6.83 (d, 4H, *J* = 8.9 Hz), 6.97 (d, 4H, *J* = 8.8 Hz), 7.96 (s, 2H) ppm; ^{13}C NMR (75 MHz, $CDCl_3$) δ = 173.7, 160.8, 159.0, 158.5, 149.2, 137.7, 136.3, 132.4, 132.2, 128.8 (4*CH), 122.9, 121.3, 120.8, 113.6 (4*CH), 110.5, 59.3, 55.3, 51.4, 35.8, 35.0, 23.5, 13.9 ppm; IR 2939, 1733, 1688, 1512, 1371, 1245, 1161,

1033, 1018, 833, 729 cm^{-1}; HRMS (ESI$^+$) calcd for $(M + H)^+$ $C_{42}H_{47}N_4O_{10}$: 767.3292, found: 767.3320; R_f = 0.12 (CH_2Cl_2/MeOH: 99/1).

Ethyl 3-(3-methoxy-3-oxopropyl)-1-(4-methoxyphenyl)-5-(pyrimidin-2-ylmethyl)-1*H*-pyrrole-2-carboxylate (5h)

^{1}H NMR (200 MHz, $CDCl_3$) δ = 1.07 (br t, 3H, *J* = 7.1 Hz, OCH_2CH_3), 2.64 (br t, 2H, *J* = 7.8 Hz), 3.11 (br t, 2H, *J* = 7.8 Hz), 3.65 (s, 3H), 3.78 (s, 3H), 4.06 (m, 4H), 5.96 (s, 1H), 6.79 (d, 2H, *J* = 8.8 Hz), 7.01 (d, 2H, *J* = 8.8 Hz), 7.08 (t, 1H, *J* = 4.9 Hz), 8.57 (d, 2H, *J* = 4.9 Hz) ppm; ^{13}C NMR (50 MHz, $CDCl_3$) δ = 173.8, 168.0, 160.8, 158.9, 157.0 (2*CH), 136.5, 132.4, 132.2, 128.9 (2*CH), 120.8, 118.6, 113.5 (2*CH), 110.4, 59.3, 55.2, 51.4, 37.3, 34.9, 23.6, 13.9 ppm; HRMS (ESI$^+$) calcd for $(M + H)^+$ $C_{23}H_{26}N_3O_5$: 424.1872, found: 424.1855; R_f = 0.13 (CH_2Cl_2/MeOH: 99/1).

Ethyl 1-(4-(allyloxy)phenyl)-3-(3-methoxy-3-oxopropyl)-5-(pyridin-2-ylmethyl)-1*H*-pyrrole-2-carboxylate (5i)

^{1}H NMR (400 MHz, $CDCl_3$) δ = 1.07 (t, 3H, *J* = 7.1 Hz, OCH_2CH_3), 2.65 (br t, 2H, *J* = 7.9 Hz), 3.11 (br t, 2H, *J* = 7.9 Hz), 3.66 (s, 3H), 3.83 (s, 2H), 4.05 (q, 2H, *J* = 7.1 Hz), 4.53 (br dt, 2H, *J* = 1.4, 5.3 Hz), 5.29 (dd, 1H, *J* = 1.4, 10.5 Hz), 5.41 (dd, 1H, *J* = 1.4, 17.2 Hz), 5.92 (s, 1H), 6.04 (m, 1H), 6.84 (d, 2H, *J* = 8.9 Hz), 6.86 (d, 1H, *J* = 7.8 Hz), 6.98 (d, 2H, *J* = 8.9 Hz), 7.07 (m, 1H), 7.50 (td, 1H, *J* = 1.8, 7.8 Hz), 8.44 (d, 1H, *J* = 4.9 Hz) ppm; ^{13}C NMR (100 MHz, $CDCl_3$) δ = 173.8 (Cq), 160.9 (Cq), 158.5 (Cq), 158.1 (Cq), 149.2 (CH), 137.7 (Cq), 136.3 (CH), 133.0 (CH), 132.6 (Cq), 132.4 (Cq), 128.9 (2*CH), 122.9 (CH), 121.4 (CH), 120.9 (Cq), 117.8 (CH_2), 114.4 (2*CH), 110.6 (CH), 68.9 (CH_2), 59.4 (CH_2), 51.5 (CH_3), 35.9 (CH_2), 35.1 (CH_2), 23.6 (CH_2), 14.0 (CH_3) ppm; IR 2982, 2950, 1734, 1695, 1570, 1512, 1435, 1370, 1240, 1169, 1081,

995, 832 cm^{-1}; HRMS (ESI^+) calcd for $(M + H)^+$ $C_{26}H_{29}N_2O_5$: 449.2076, found: 449.2075; R_f = 0.22 (CH_2Cl_2/MeOH: 99/1).

Ethyl 1-(4-(benzyloxy)phenyl)-3-(3-methoxy-3-oxopropyl)-5-(quinolin-2-ylmethyl)-1*H*-pyrrole-2-carboxylate (5j)

^{1}H NMR (400 MHz, $CDCl_3$) δ = 1.08 (br t, 3H, *J* = 7.1 Hz, OCH_2CH_3), 2.65 (br t, 2H, *J* = 7.8 Hz), 3.13 (br t, 2H, *J* = 7.8 Hz), 3.66 (s, 3H), 4.04 (s, 2H), 4.07 (q, 2H, *J* = 7.1 Hz), 5.02 (s, 2H), 5.95 (s, 1H), 6.89 (d, 2H, *J* = 8.9 Hz), 7.02 (d, 2H, *J* = 8.9 Hz), 7.09 (d, 1H, *J* = 8.5 Hz), 7.32–7.45 (m, 5H), 7.50 (m, 1H), 7.67 (m, 1H), 7.76 (br d, 1H, *J* = 8.5 Hz), 7.96 (br d, 1H, *J* = 8.5 Hz), 8.00 (br d, 1H, *J* = 8.5 Hz) ppm; ^{13}C NMR (100 MHz, $CDCl_3$) δ = 173.7, 160.9, 158.7, 158.6, 158.3, 147.7, 137.5, 136.6, 136.3, 132.6, 132.4, 129.4, 129.0, 128.9, 128.5, 128.0, 127.4, 126.7, 126.0, 121.1, 121.0, 114.5, 110.7, 70.1, 59.4, 51.4, 36.8, 35.0, 23.6, 14.0 ppm; IR 2892, 1732, 1694, 1510, 1451, 1368, 1295, 1239, 1166, 1080, 1022, 829, 736, 698 cm^{-1}; R_f = 0.3 (CH_2Cl_2/MeOH: 99/1).

Ethyl 5-(hydroxy(pyridin-3-yl)methyl)-3-(3-methoxy-3-oxopropyl)-1-(4-methoxyphenyl)-1*H*-pyrrole-2-carboxylate (9b)

^{1}H NMR (200 MHz, $CDCl_3$) δ = 1.08 (br t, 3H, *J* = 7.1 Hz, OCH_2CH_3), 2.62 (br t, 2H, *J* = 7.8 Hz), 3.09 (m, 2H), 3.65 (s, 3H), 3.82 (s, 3H), 4.06 (q, 2H, *J* = 7.1 Hz), 5.48 (s, 1H), 6.04 (s, 1H), 6.84 (m, 2H), 6.91 (dd, 1H, *J* = 2.4, 8.7 Hz), 7.17 (dd, 1H, *J* = 1.9, 8.7 Hz), 7.22 (dd, 1H, *J* = 4.9, 7.7 Hz), 7.57 (d, 1H, *J* = 7.9 Hz), 8.28 (broad s, 1H), 8.44 (br s, 1H) ppm; ^{13}C NMR (50 MHz, $CDCl_3$) δ = 173.7, 160.8, 159.4, 148.7, 148.0, 140.8, 134.5, 132.1, 131.5, 129.3, 129.0, 123.4, 121.9, 114.0, 113.7, 109.7, 66.8, 59.8, 55.5, 51.6, 35.0, 23.5, 14.0 ppm; IR 3443, 2933, 1733, 1693, 1512, 1440,

1246, 1158, 1082, 1028, 835, 734 cm^{-1}; HRMS (ESI$^+$) calcd for $(M + H)^+$ $C_{24}H_{27}N_2O_6$: 439.1869, found: 439.1860; R_f = 0.3 (CH_2Cl_2/MeOH: 95/5).

Ethyl 5-(hydroxy(pyridin-4-yl)methyl)-3-(3-methoxy-3-oxopropyl)-1-(4-methoxyphenyl)-1*H*-pyrrole-2-carboxylate (9c)

^{1}H NMR (200 MHz, $CDCl_3$) δ = 1.10 (br t, 3H, *J* = 7.1 Hz, OCH_2CH_3), 2.61 (br t, 2H, *J* = 8.1 Hz), 3.08 (br t, 2H, *J* = 8.1 Hz), 3.65 (s, 3H), 3.84 (s, 3H), 4.07 (q, 2H, *J* = 7.1 Hz), 5.47 (s, 1H), 5.95 (s, 1H), 6.87 (d, 2H, *J* = 8.9 Hz), 7.03 (m, 1H), 7.09–7.23 (m, 3H), 8.51 (br s, 2H) ppm; ^{13}C NMR (50 MHz, $CDCl_3$) δ = 173.6, 160.7, 159.4, 151.5, 149.0, 140.1, 132.0, 131.4, 129.3, 129.1, 122.1, 121.6, 113.8, 110.1, 67.2, 59.8, 55.5, 51.5, 34.9, 23.4, 13.9 ppm; HRMS (ESI$^+$) calcd for $(M + H)^+$ $C_{24}H_{27}N_2O_6$: 439.1869, found: 439.1853; R_f = 0.17 (CH_2Cl_2/MeOH: 99/1).

Ethyl 5-(hydroxy(quinolin-2-yl)methyl)-3-(3-methoxy-3-oxopropyl)-1-(4-methoxyphenyl)-1*H*-pyrrole-2-carboxylate (9d)

^{1}H NMR (300 MHz, $CDCl_3$) δ = 1.08 (br t, 3H, *J* = 7.1 Hz, OCH_2CH_3), 2.60 (br t, 2H, *J* = 7.9 Hz), 3.07 (br t, 2H, *J* = 7.9 Hz), 3.62 (s, 3H), 3.73 (s, 3H), 4.06 (q, 2H, *J* = 7.1 Hz), 5.60 (s, 1H), 5.89 (s, 1H), 6.60 (dd, 1H, *J* = 2.5, 8.6 Hz), 6.91 (dd, 1H, *J* = 1.9, 8.2 Hz), 6.98 (dd, 1H, *J* = 2.1, 8.6 Hz), 7.11 (d, 1H, *J* = 8.5 Hz), 7.27 (dd, 1H, *J* = 2.4, 8.4 Hz), 7.55 (t, 1H, *J* = 7.5 Hz), 7.72 (br t, 1H, *J* = 7.6 Hz), 7.81 (d, 1H, *J* = 7.9 Hz), 7.98 (d, 1H, *J* = 8.6 Hz), 8.06 (d, 1H, *J* = 8.6 Hz) ppm; ^{13}C NMR (75 MHz, $CDCl_3$) δ = 173.7, 160.9, 159.1, 158.4, 145.9, 140.4, 136.7, 132.1, 131.6, 129.8, 129.4, 129.3, 128.7, 127.5, 127.5, 126.7, 121.9, 119.1, 113.8, 112.9, 110.7, 59.6, 55.3, 51.4, 35.0, 23.5, 13.9 ppm; IR 3377, 2946, 1733, 1690, 1512, 1371, 1297, 1245, 1161, 1081, 1031, 832, 728 cm^{-1}; HRMS (ESI$^+$) calcd for $(M - H)^+$ $C_{28}H_{27}N_2O_6$: 487.1869, found: 487.1872; R_f = 0.3 (CH_2Cl_2/MeOH: 98/2).

Ethyl 5-((3-chloropyridin-2-yl)(hydroxy)methyl)-3-(3-methoxy-3-oxopropyl)-1-(4-methoxyphenyl)-1*H*-pyrrole-2-carboxylate (9e)

^{1}H NMR (300 MHz, $CDCl_3$) δ = 1.10 (t, 3H, *J* = 7.1 Hz, OCH_2CH_3), 2.56 (t, 2H, *J* = 7.9 Hz), 3.02 (m, 2H), 3.61 (s, 3H), 3.85 (s, 3H), 4.08 (q, 2H, *J* = 7.1 Hz), 5.45 (s, 1H), 5.50 (s, 0.56H), 6.96 (d, 2H, *J* = 8.6 Hz), 7.20–7.31 (m, 2H), 7.38 (m, 1H), 7.65 (dd, 1H, *J* = 1.3, 8.0 Hz), 8.48 (dd, 1H, *J* = 1.1, 4.7 Hz) ppm; ^{13}C NMR (75 MHz, $CDCl_3$) δ = 173.7, 160.9, 159.3, 155.7, 145.7, 139.8, 137.5, 132.0, 131.7, 129.9, 129.6, 129.0, 123.9, 121.5, 113.8, 113.4, 109.1, 64.1, 59.6, 55.4, 51.4, 35.0, 23.6, 14.0 ppm; IR 3436, 2920, 1736, 1700, 1513, 1444, 1370, 1248, 1177, 1083, 834 cm^{-1}; HRMS (ESI^+) calcd for $(M + H)^+$ $C_{24}H_{26}N_2O_6{}^{35}Cl$: 473.1479, found: 473.1476; R_f = 0.15 (CH_2Cl_2/MeOH: 99/1).

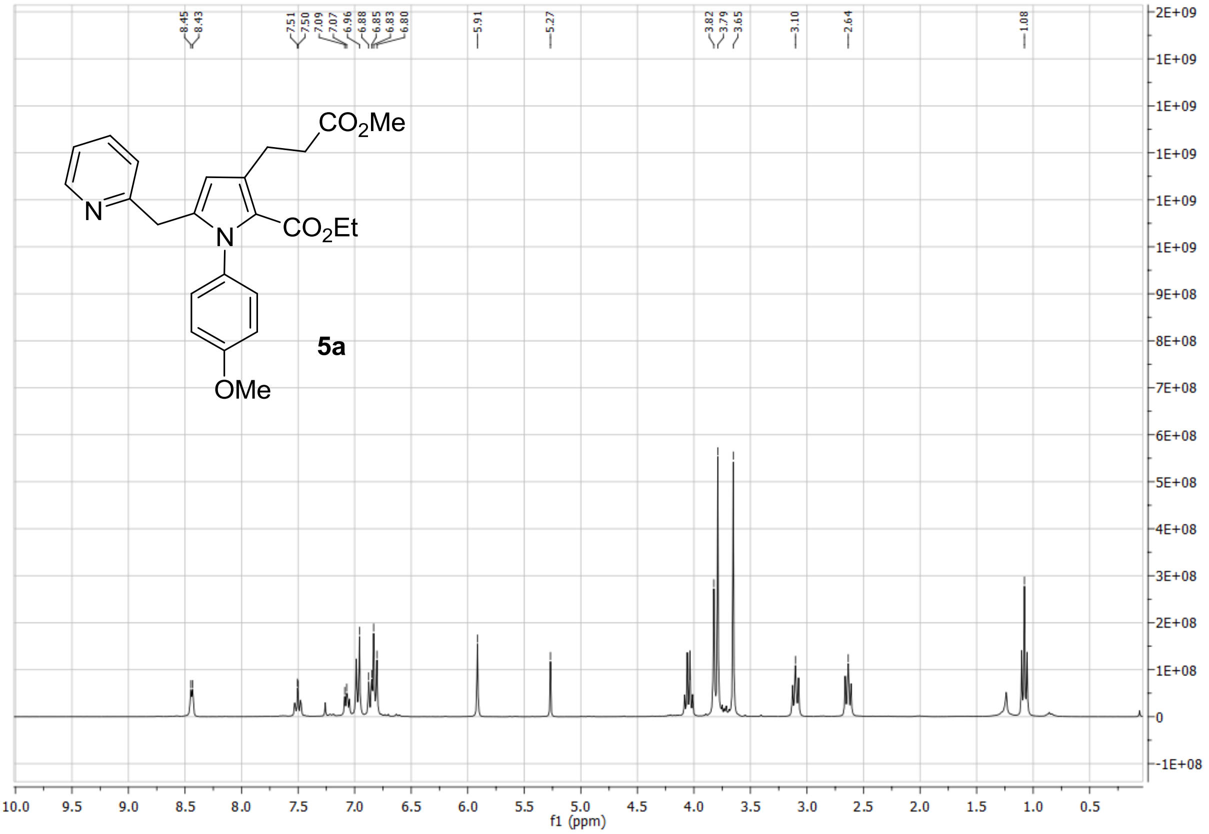
8.45
8.43
7.51
7.50
7.09
7.07
6.96
6.88
6.85
6.83
6.80
5.91
5.27
3.82
3.79
3.65
3.10
2.64
1.08
CO2Me
N
N
CO2Et
OMe
5a
f1 (ppm)

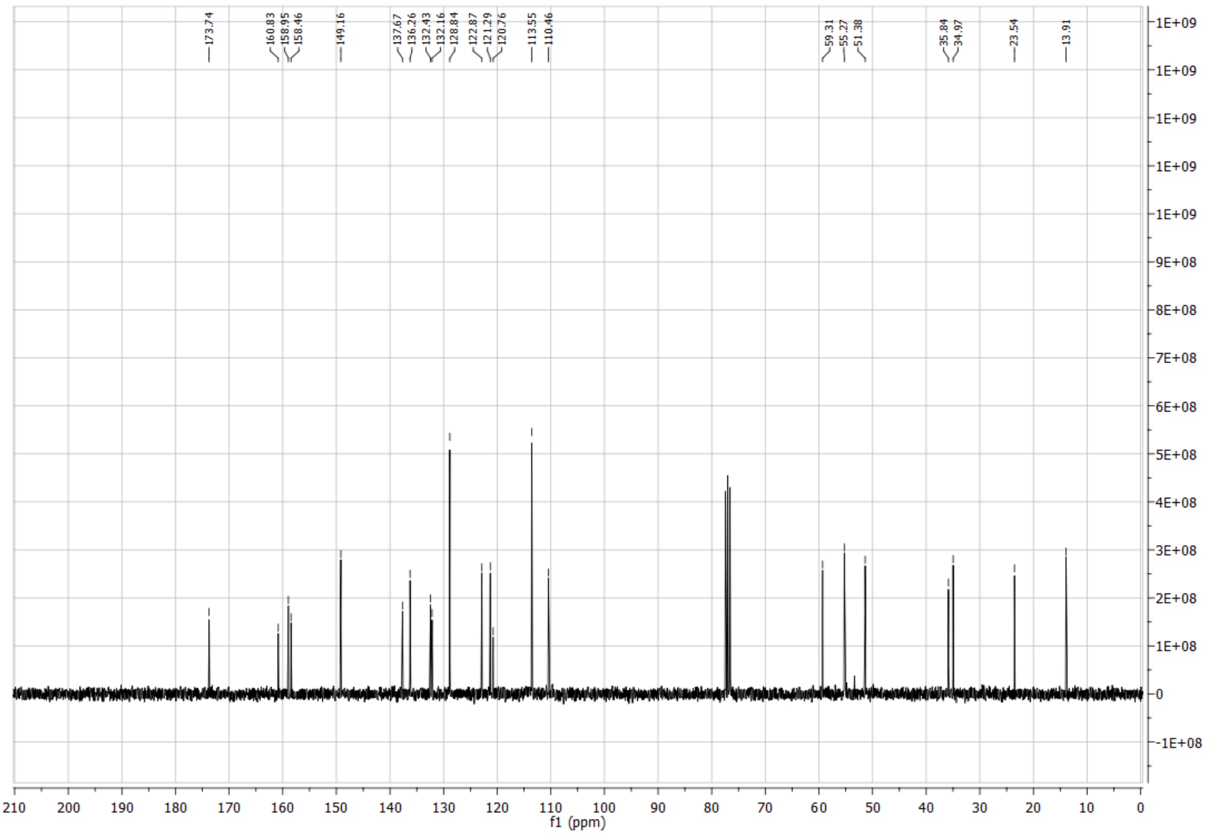
173.74
160.83
158.95
158.46
149.16
137.67
136.26
132.43
132.16
128.84
122.87
121.29
120.76
113.55
110.46
59.31
55.27
51.38
35.84
34.97
23.54
13.91
f1 (ppm)

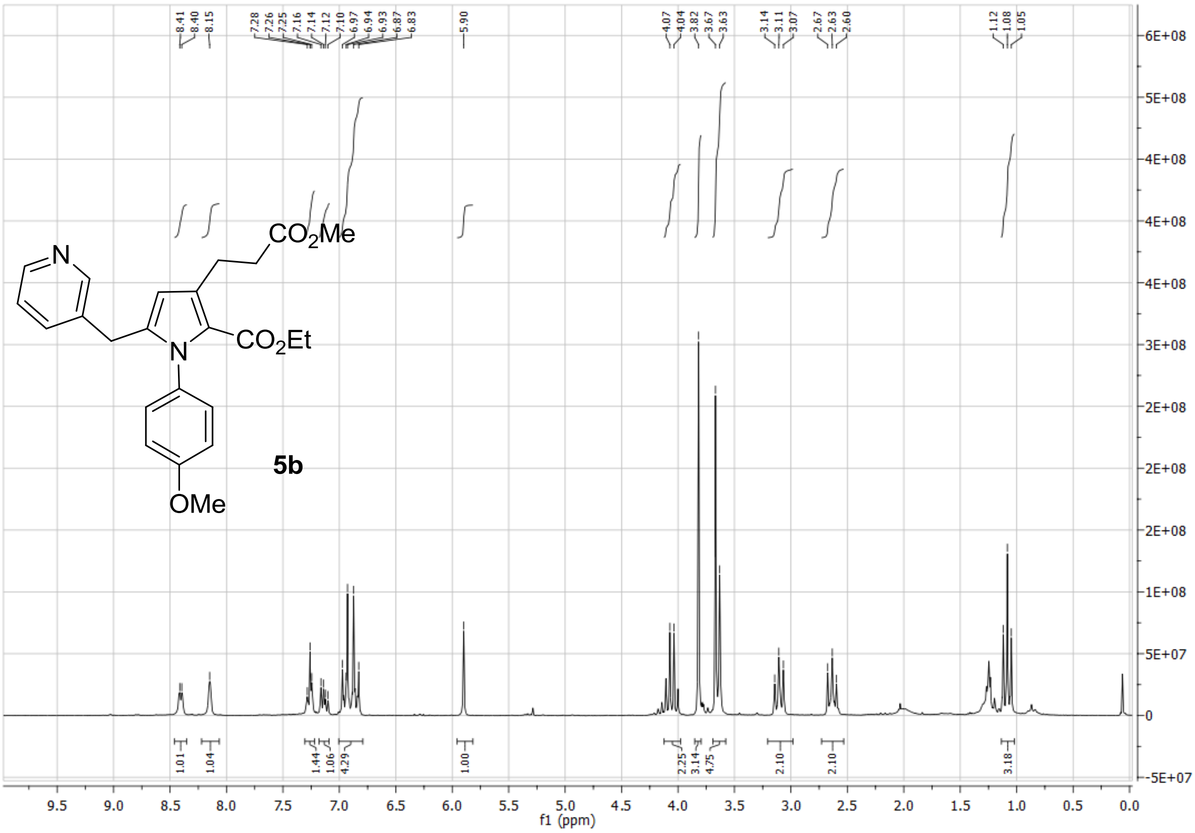
CO2Me
N
CO2Et
N
OMe
5b
f1 (ppm)

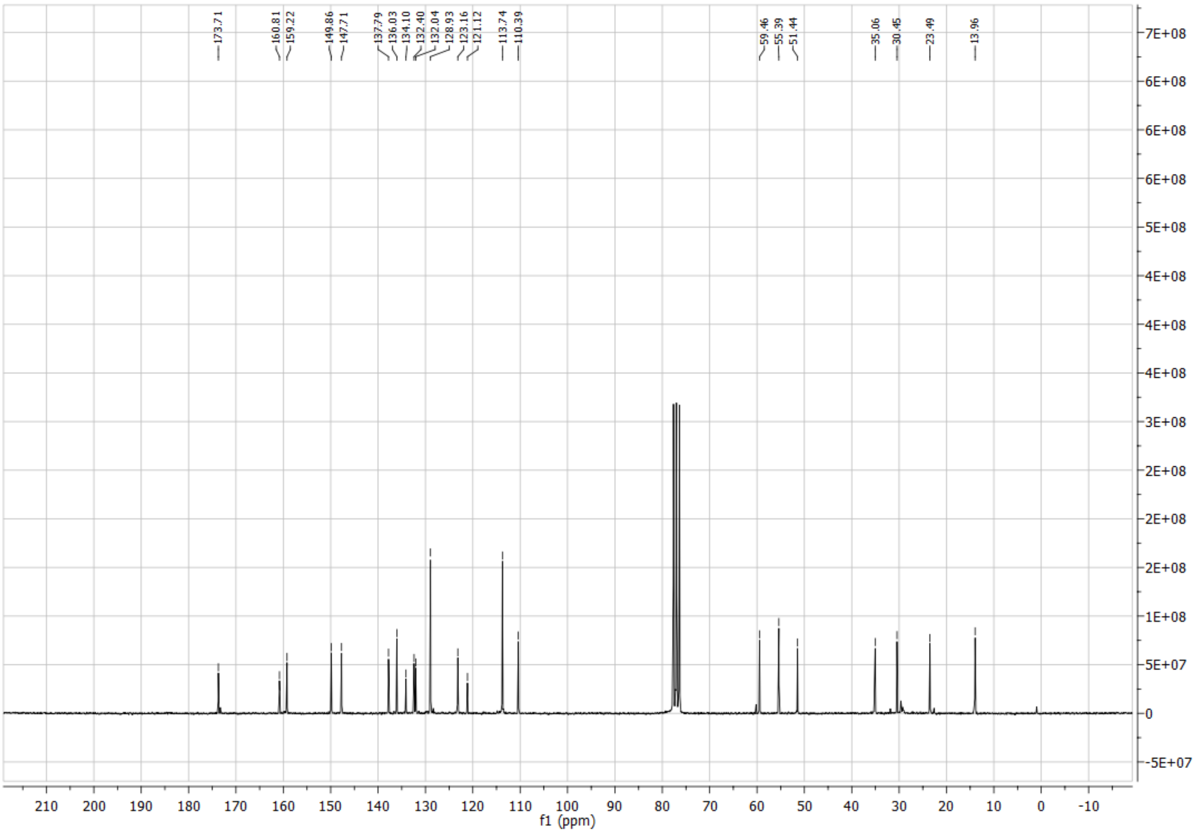
f1 (ppm)

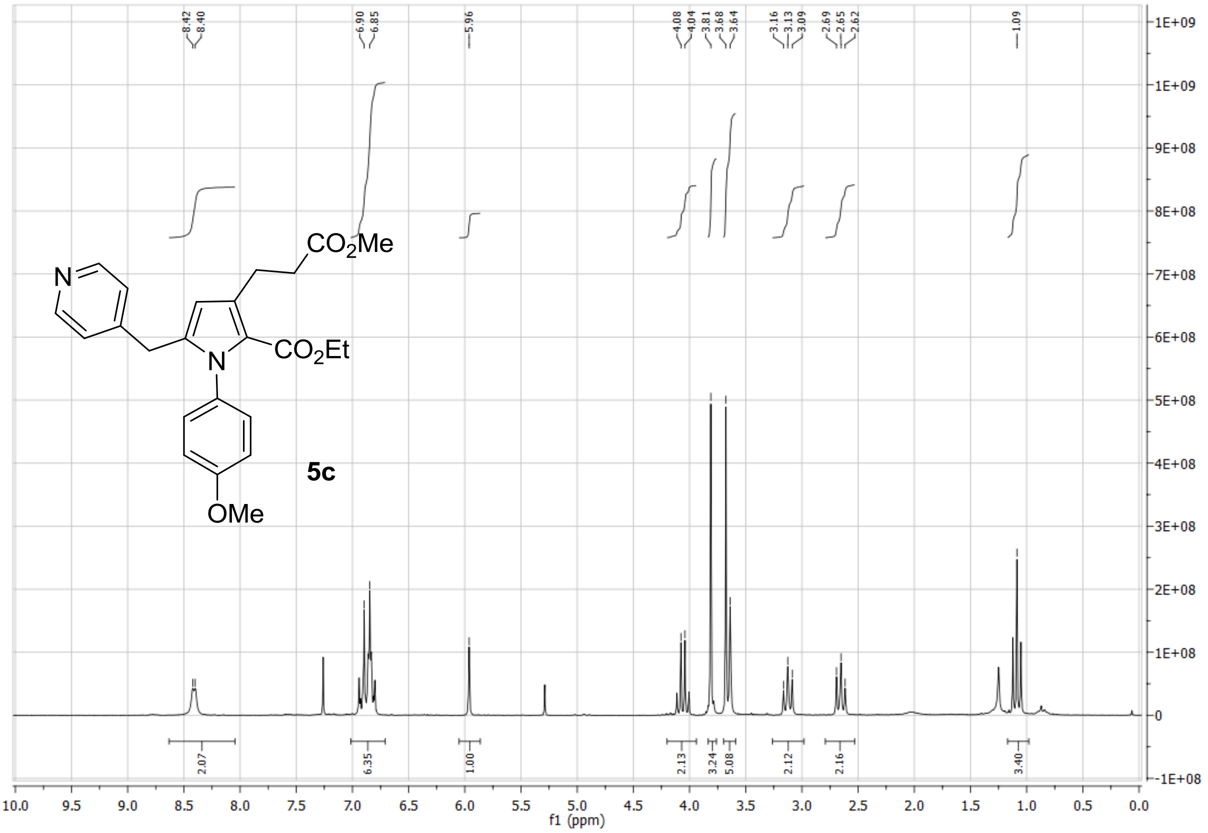
5c
f1 (ppm)

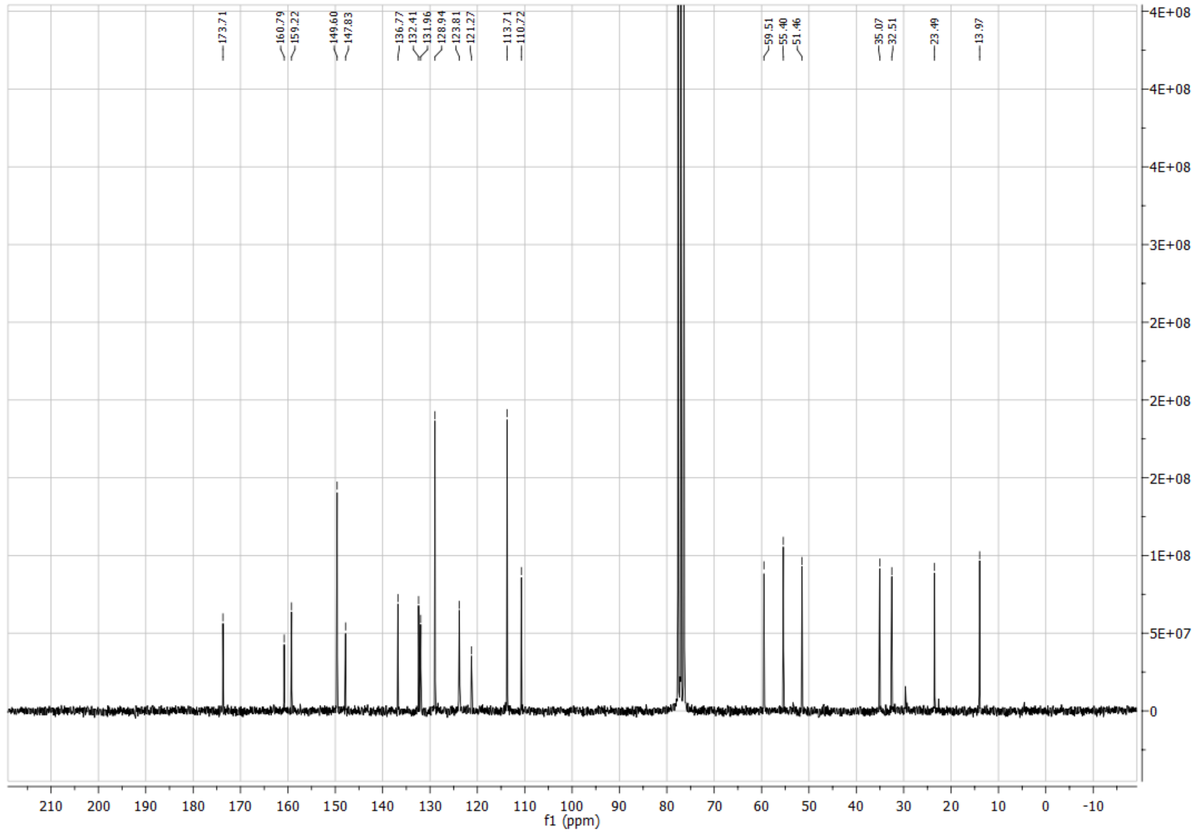
f1 (ppm)

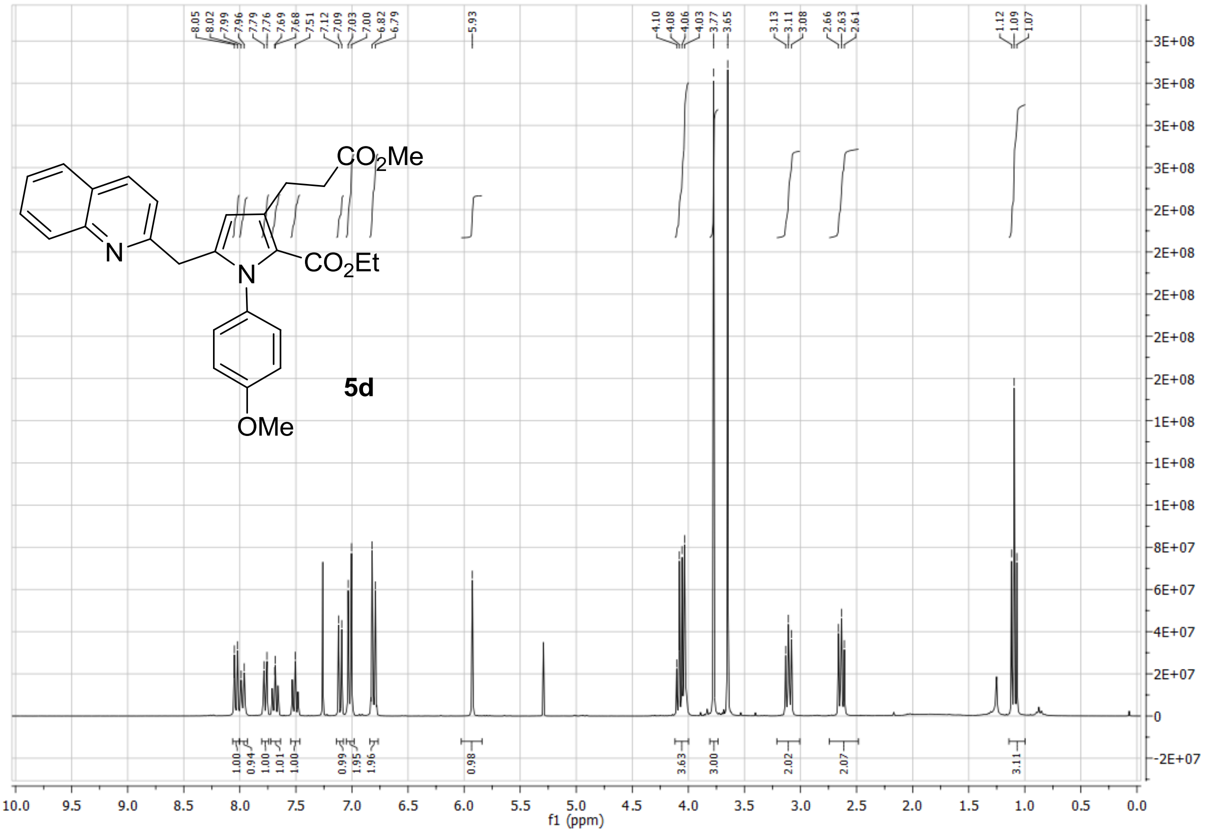
8.05 8.02 7.99 7.96 7.79 7.76 7.69 7.68 7.51 7.12 7.09 7.03 7.00 6.82 6.79
5.93
4.10 4.08 4.06 4.03 3.77 3.65
3.13 3.11 3.08 2.66 2.63 2.61
1.12 1.09 1.07
CO2Me
N
N
CO2Et
OMe
5d
1.00 0.94 1.00 1.01 1.00 0.99 1.95 1.96 0.98 3.63 3.00 2.02 2.07 3.11
f1 (ppm)

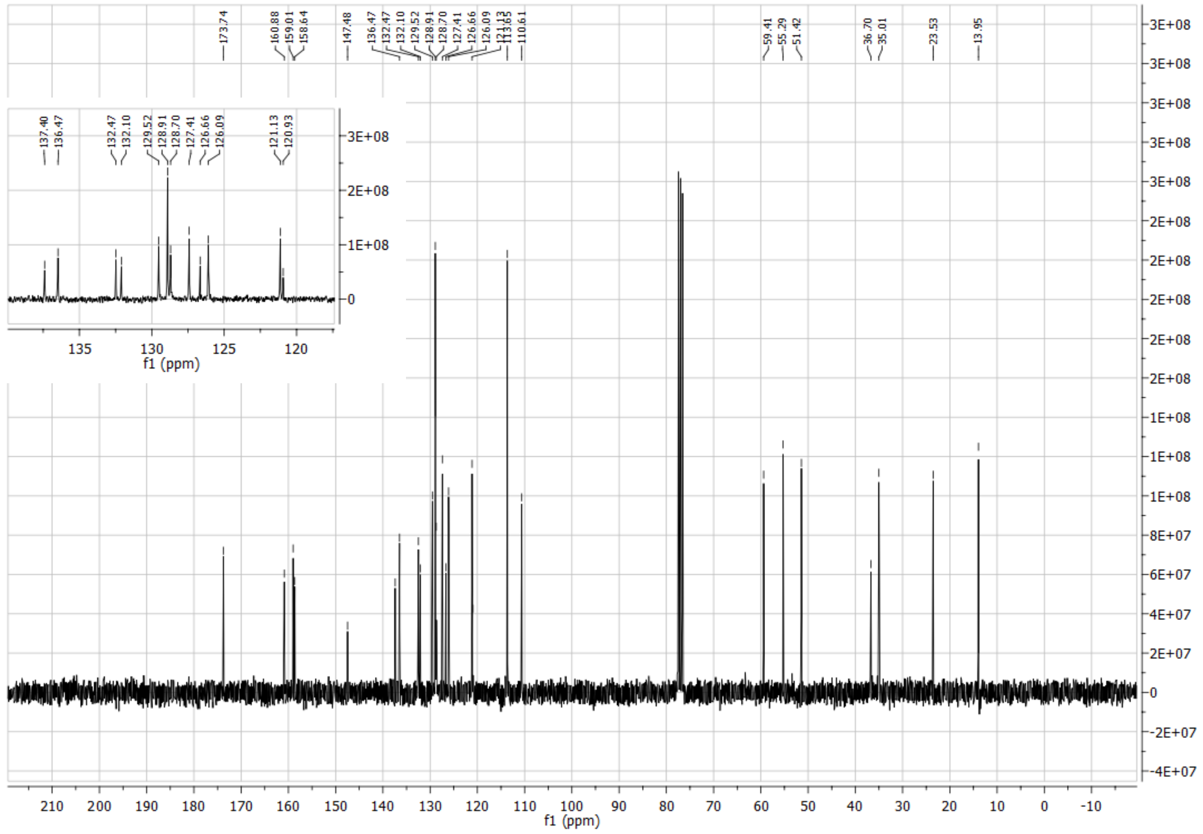
173.74
160.88 159.01 158.64
147.48
136.47 132.47 132.10 129.52 128.91 128.70 127.41 126.66 126.09 121.13 113.65 110.61
59.41 55.29 51.42
36.70 35.01
23.53
13.95
137.40 136.47 132.47 132.10 129.52 128.91 128.70 127.41 126.66 126.09 121.13 120.93
f1 (ppm)

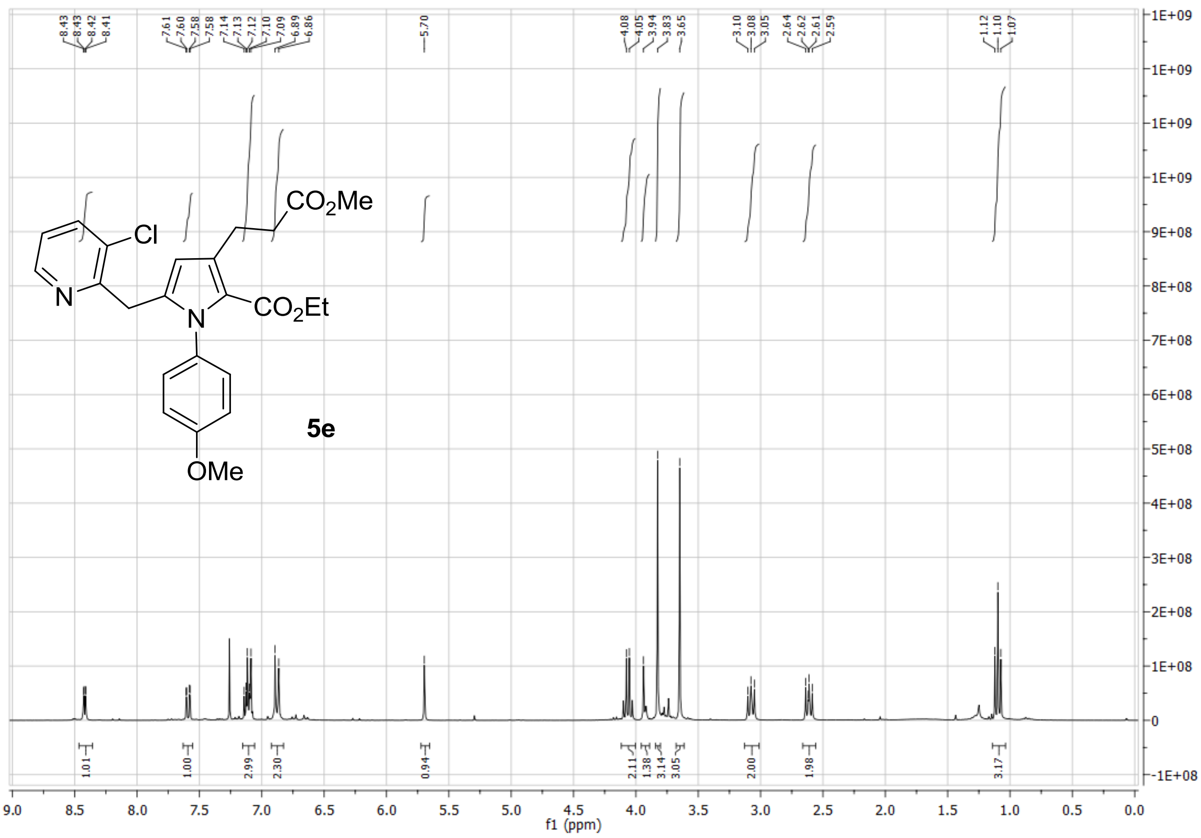
CO2Me
Cl
N
N
CO2Et
OMe
5e
f1 (ppm)

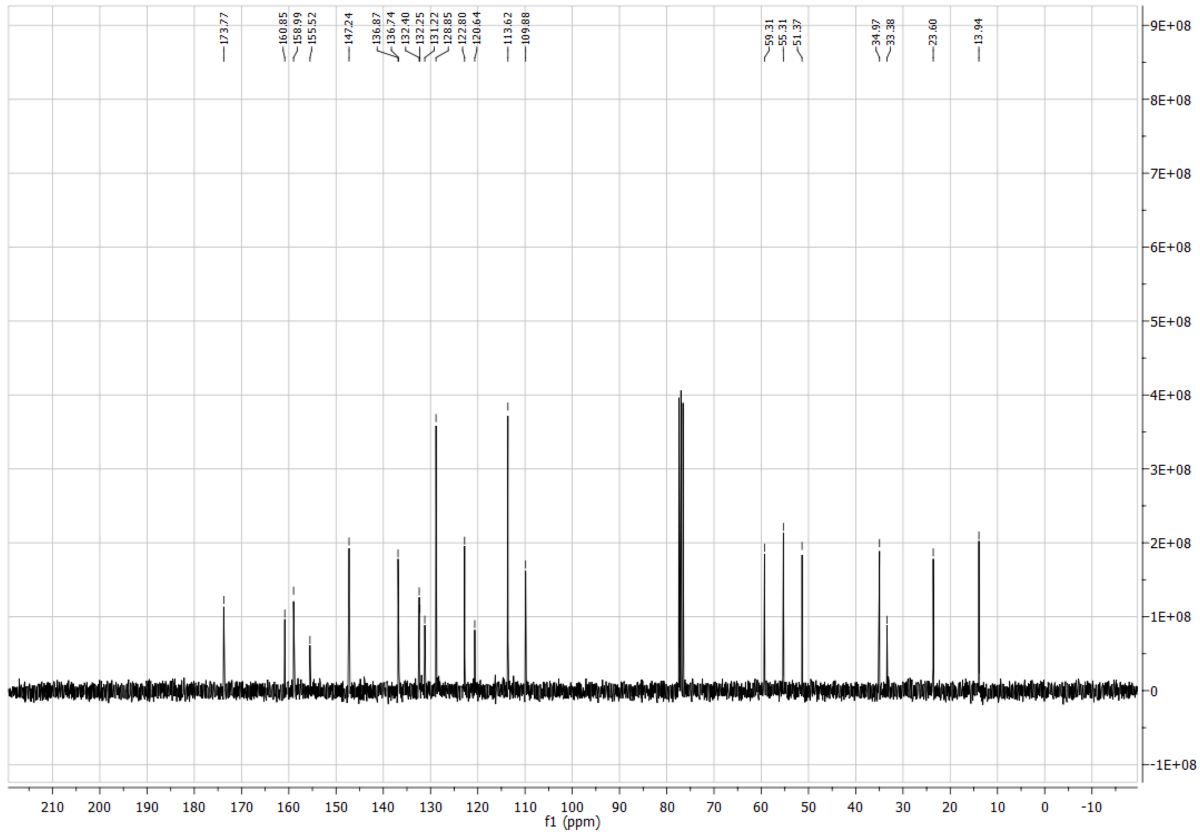
f1 (ppm)

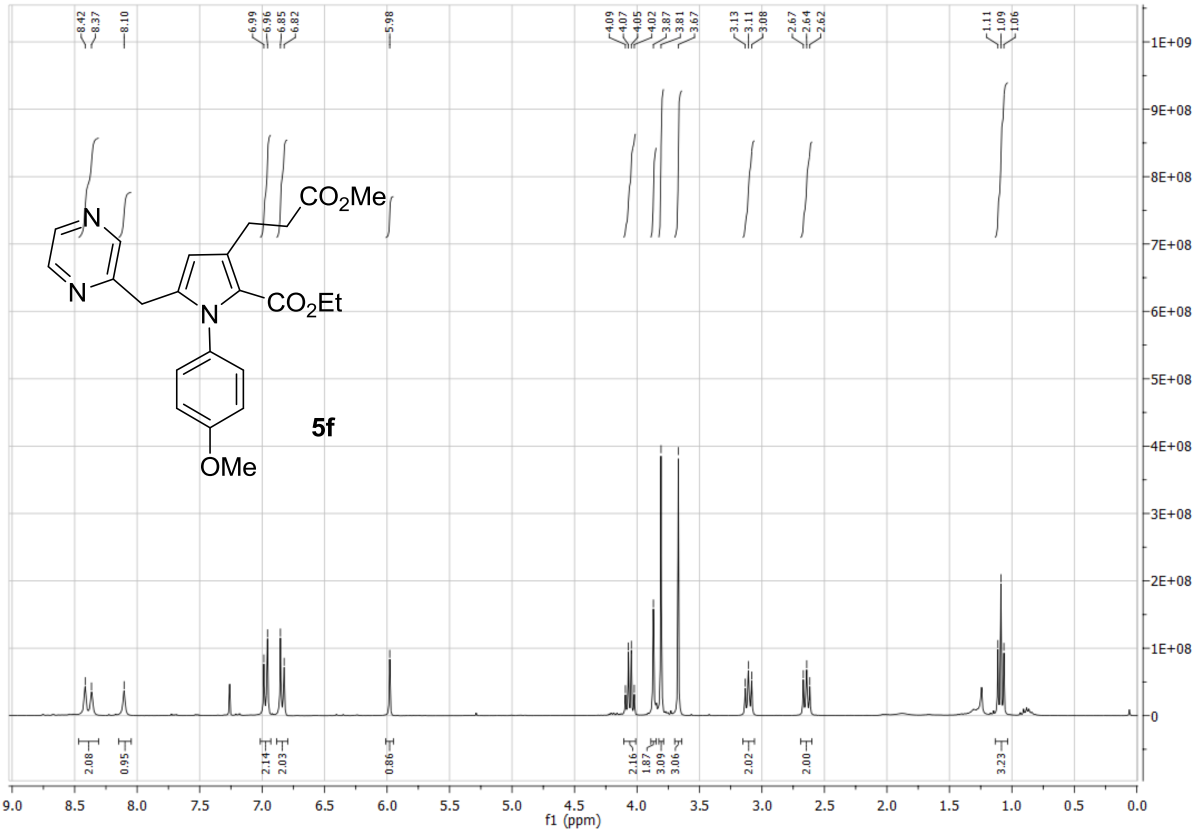

5f

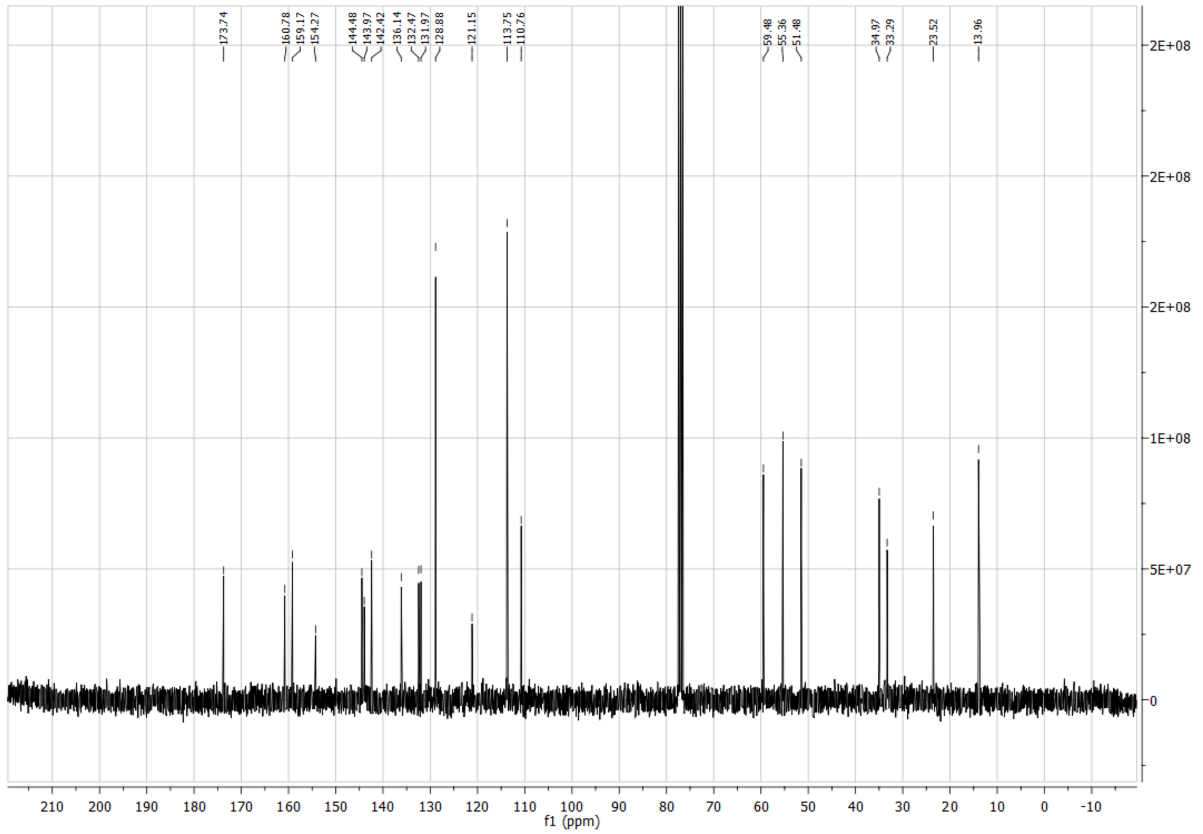

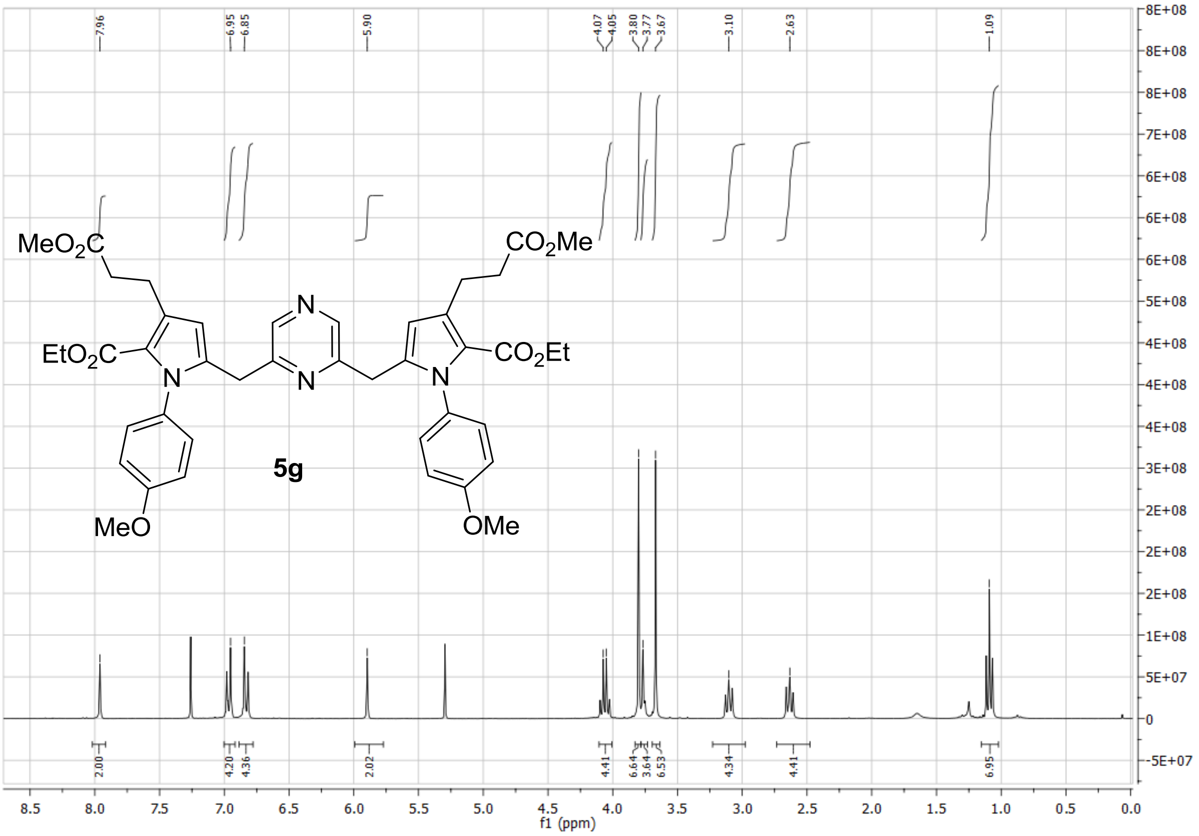
5g
MeO2C
CO2Me
EtO2C
CO2Et
MeO
OMe
7.96
6.95
6.85
5.90
4.07
4.05
3.80
3.77
3.67
3.10
2.63
1.09
2.00
4.20
4.36
2.02
4.41
6.64
3.64
6.53
4.34
4.41
6.95
f1 (ppm)

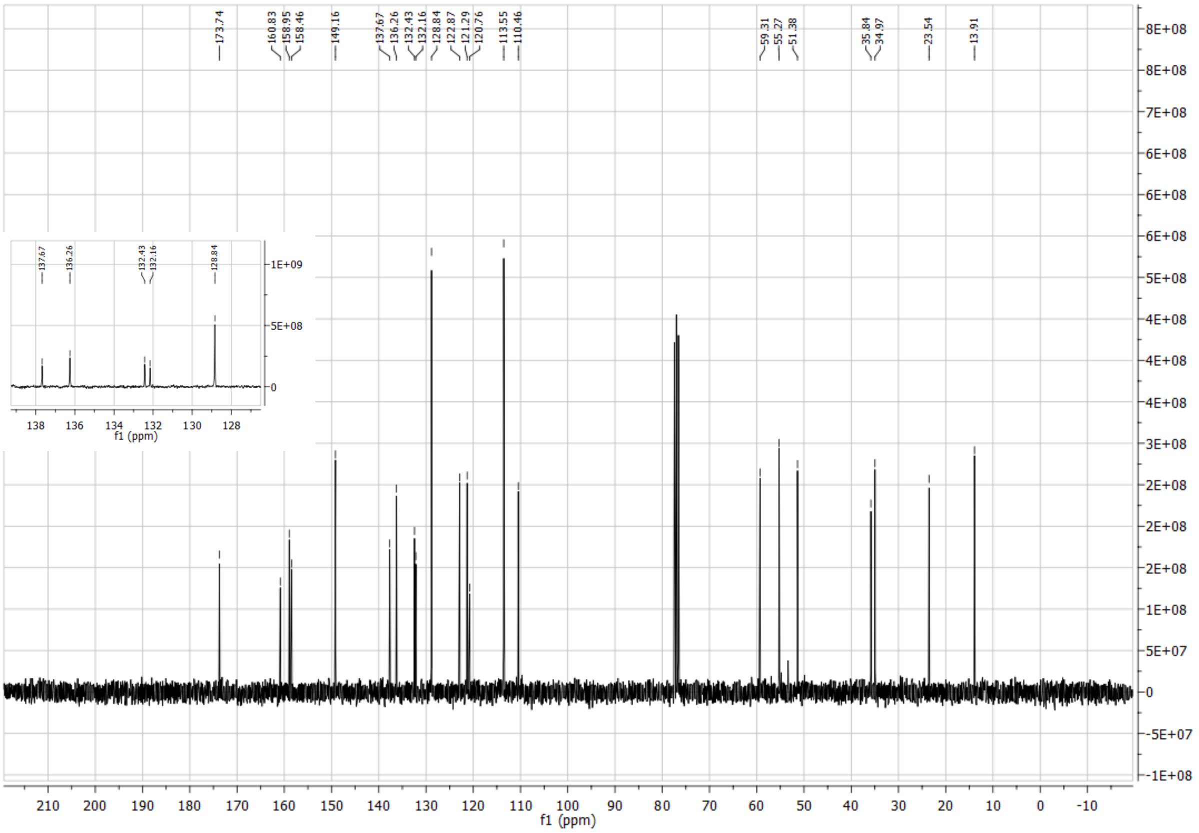
173.74
160.83
158.95
158.46
149.16
137.67
136.26
132.43
132.16
128.84
122.87
121.29
120.76
113.55
110.46
59.31
55.27
51.38
35.84
34.97
23.54
13.91
f1 (ppm)

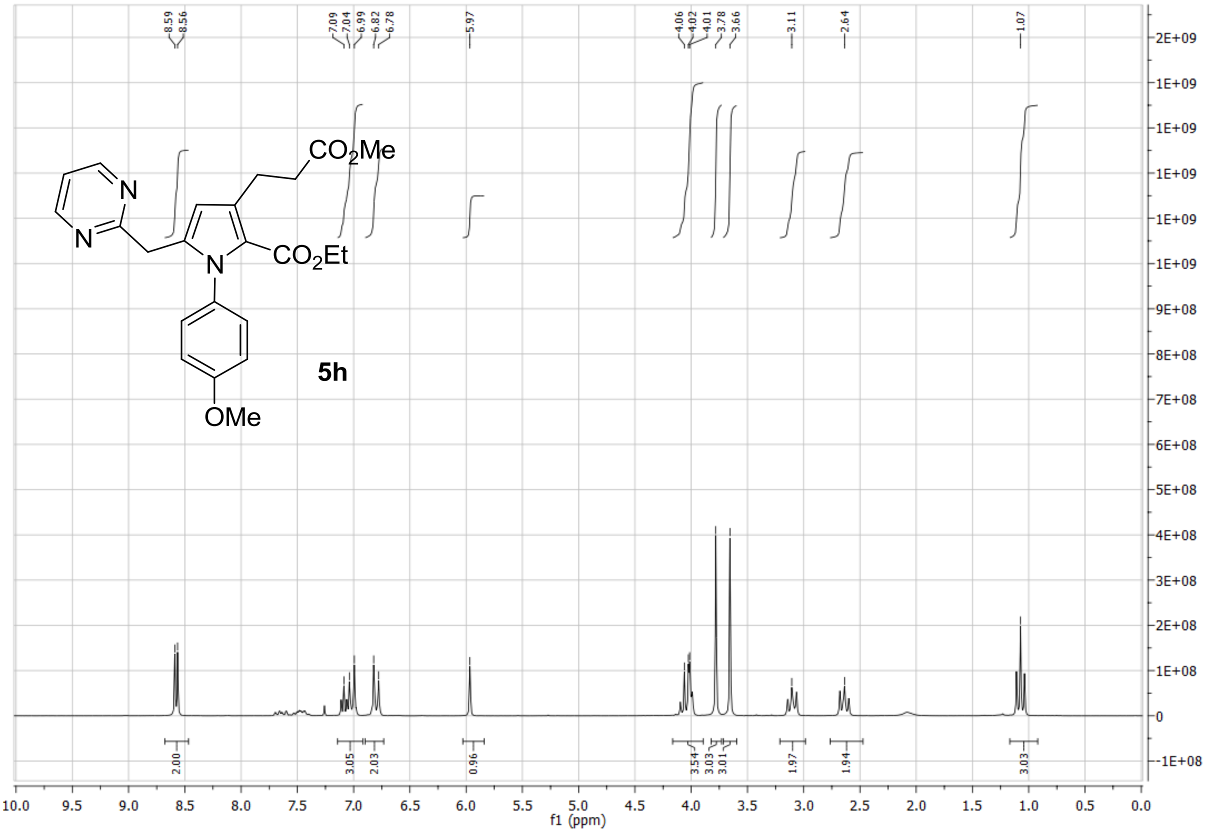
CO2Me
CO2Et
N
N
N
OMe
5h
f1 (ppm)

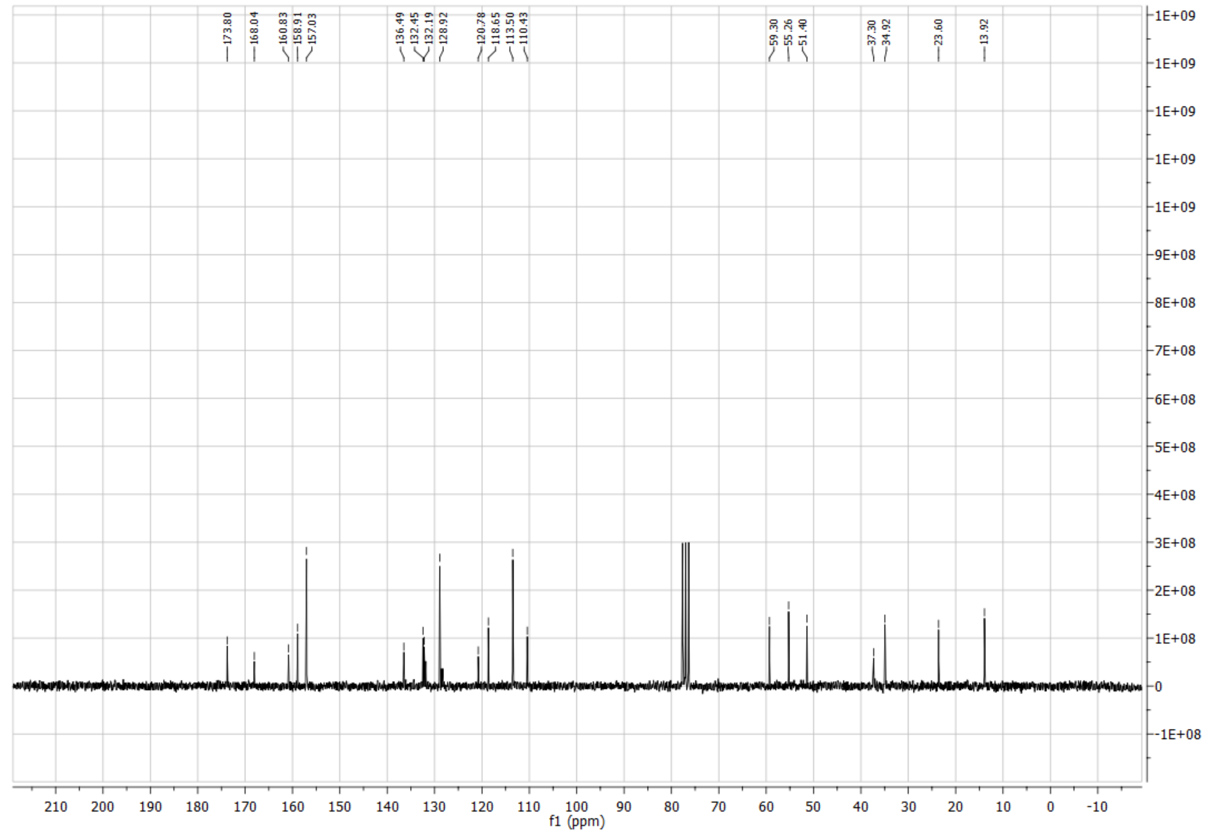
f1 (ppm)

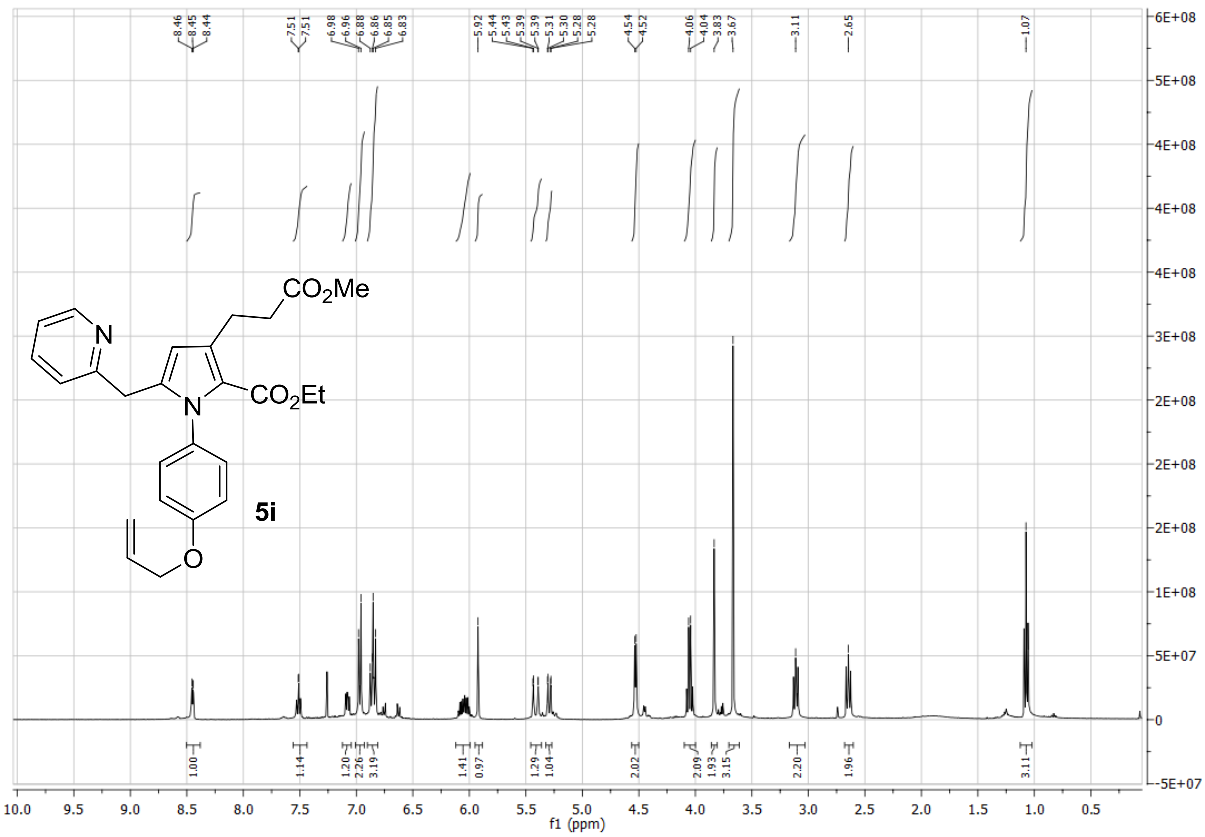
8.46 8.45 8.44 7.51 7.51 6.98 6.96 6.88 6.86 6.85 6.83 5.92 5.44 5.43 5.39 5.39 5.31 5.30 5.28 5.28 4.54 4.52 4.06 4.04 3.83 3.67 3.11 2.65 1.07
CO2Me
N
CO2Et
N
O
5i
1.00 1.14 1.20 2.26 3.19 1.41 0.97 1.29 1.04 2.02 2.09 1.93 3.15 2.20 1.96 3.11
f1 (ppm)

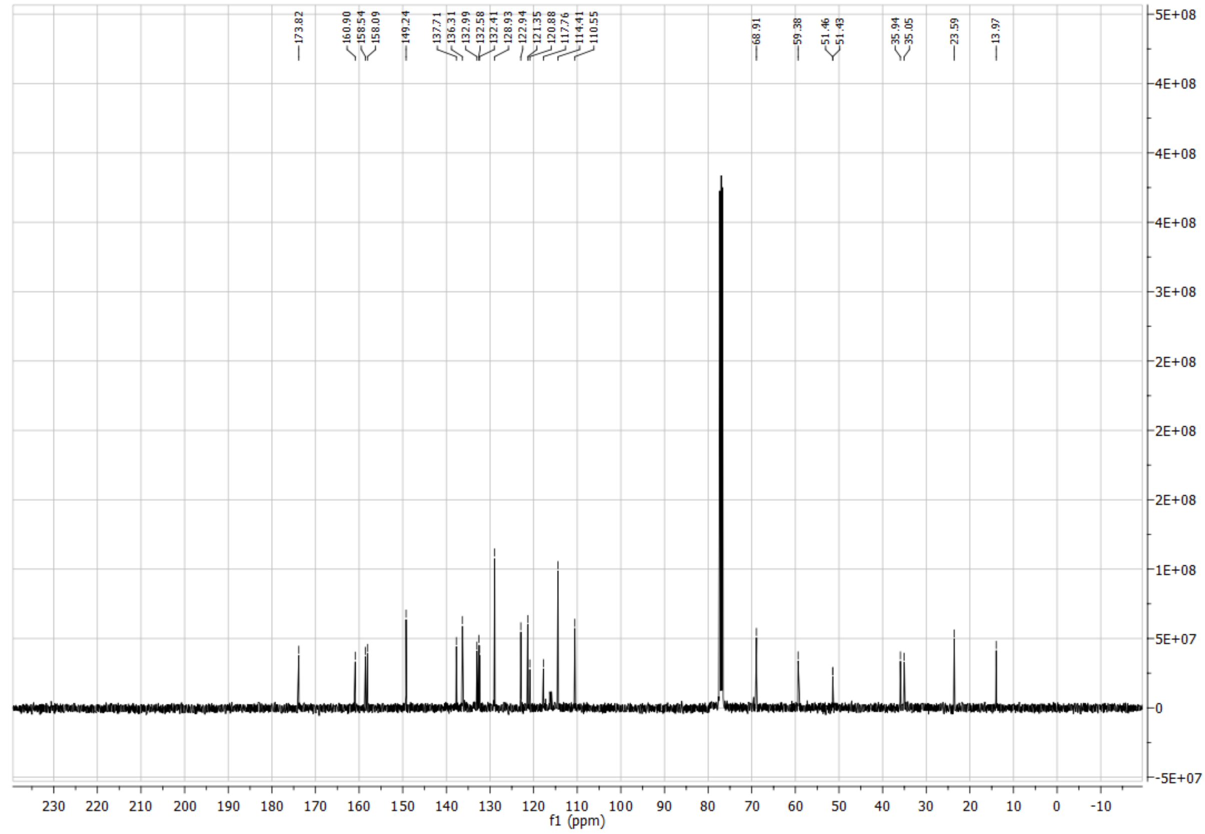
173.82 160.90 158.54 158.09 149.24 137.71 136.31 132.99 132.58 132.41 128.93 122.94 121.35 120.88 117.76 114.41 110.55 68.91 59.38 51.46 51.43 35.94 35.05 23.59 13.97
f1 (ppm)

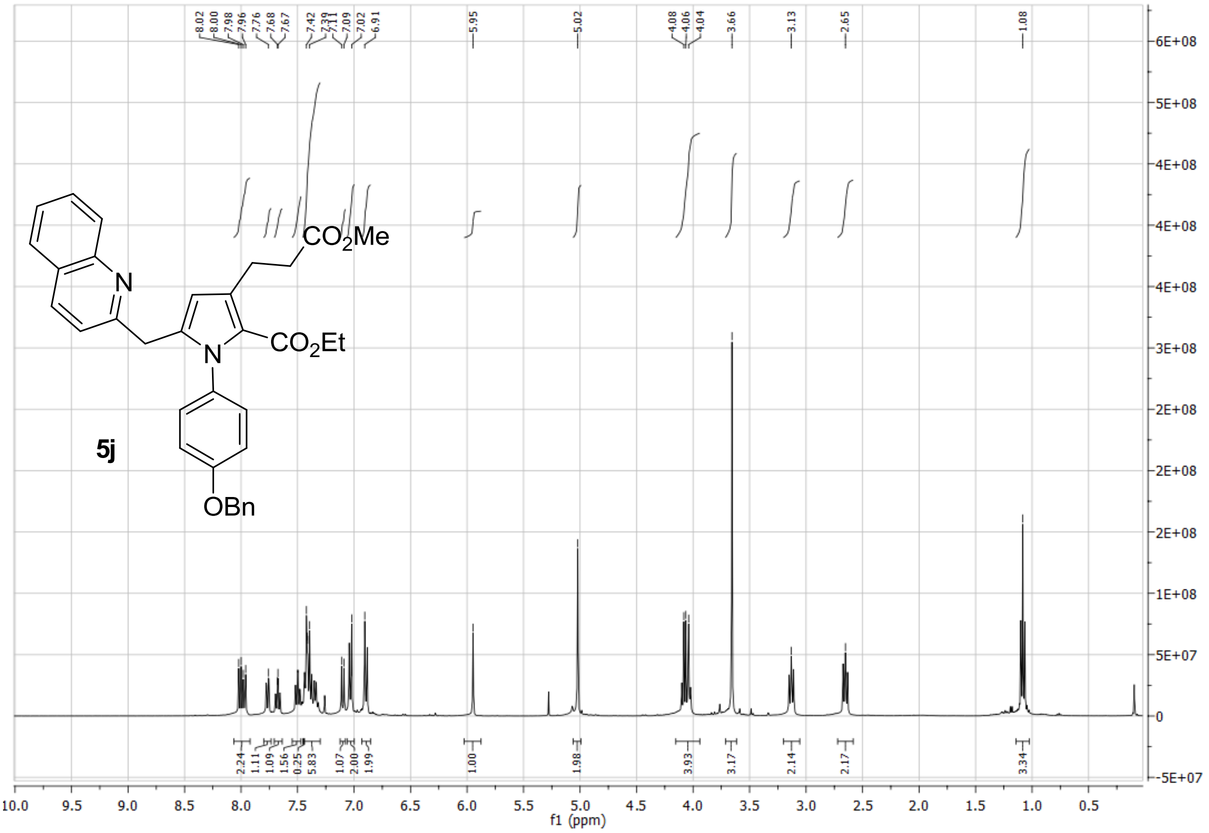

5j

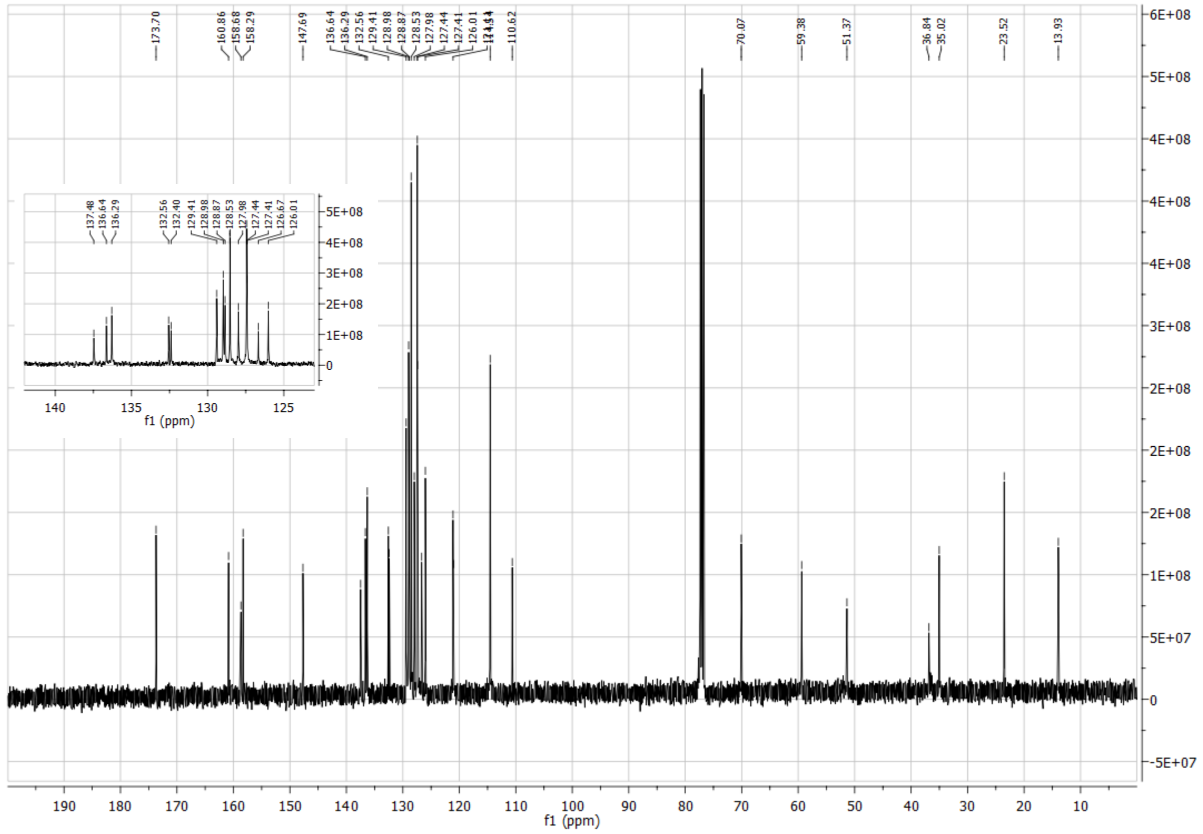

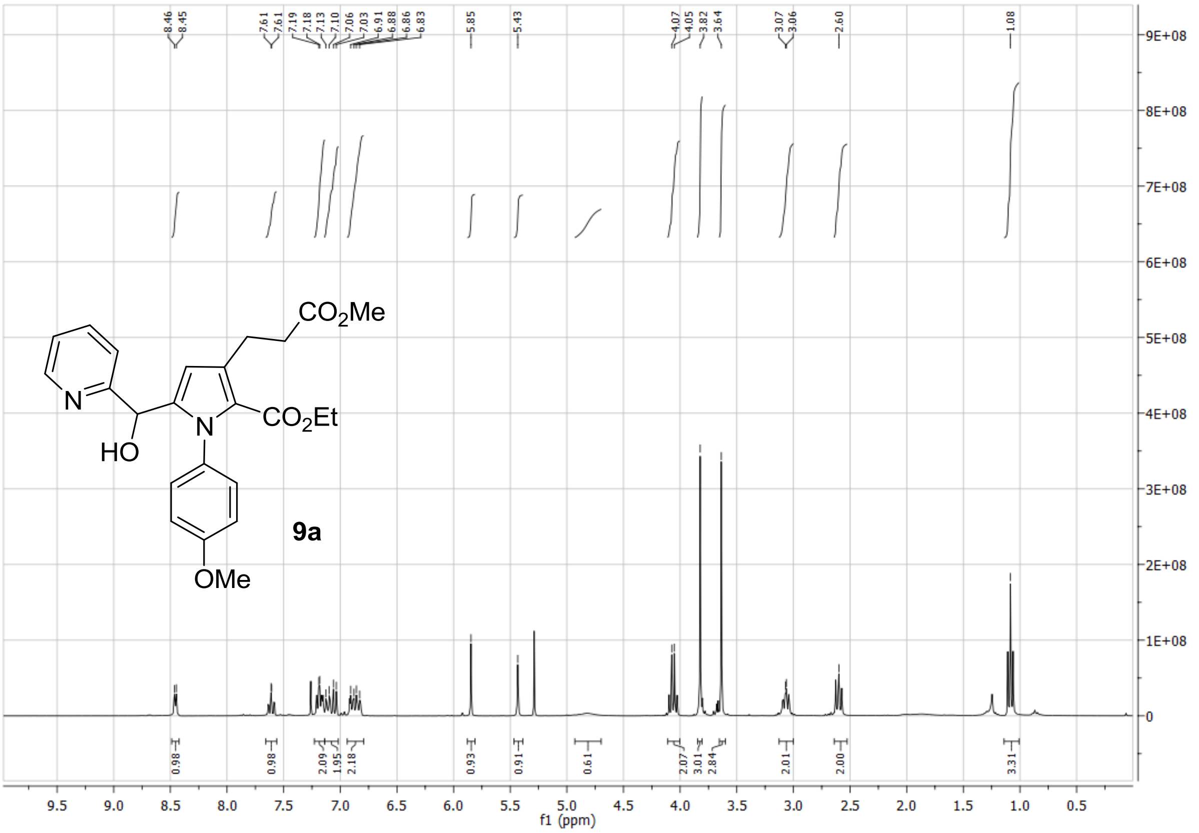
8.46
8.45
7.61
7.61
7.19
7.18
7.13
7.10
7.06
7.03
6.91
6.88
6.86
6.83
5.85
5.43
4.07
4.05
3.82
3.64
3.07
3.06
2.60
1.08
CO2Me
N
CO2Et
N
HO
OMe
9a
0.98
0.98
2.09
1.95
2.18
0.93
0.91
0.61
2.07
3.01
2.84
2.01
2.00
3.31
f1 (ppm)

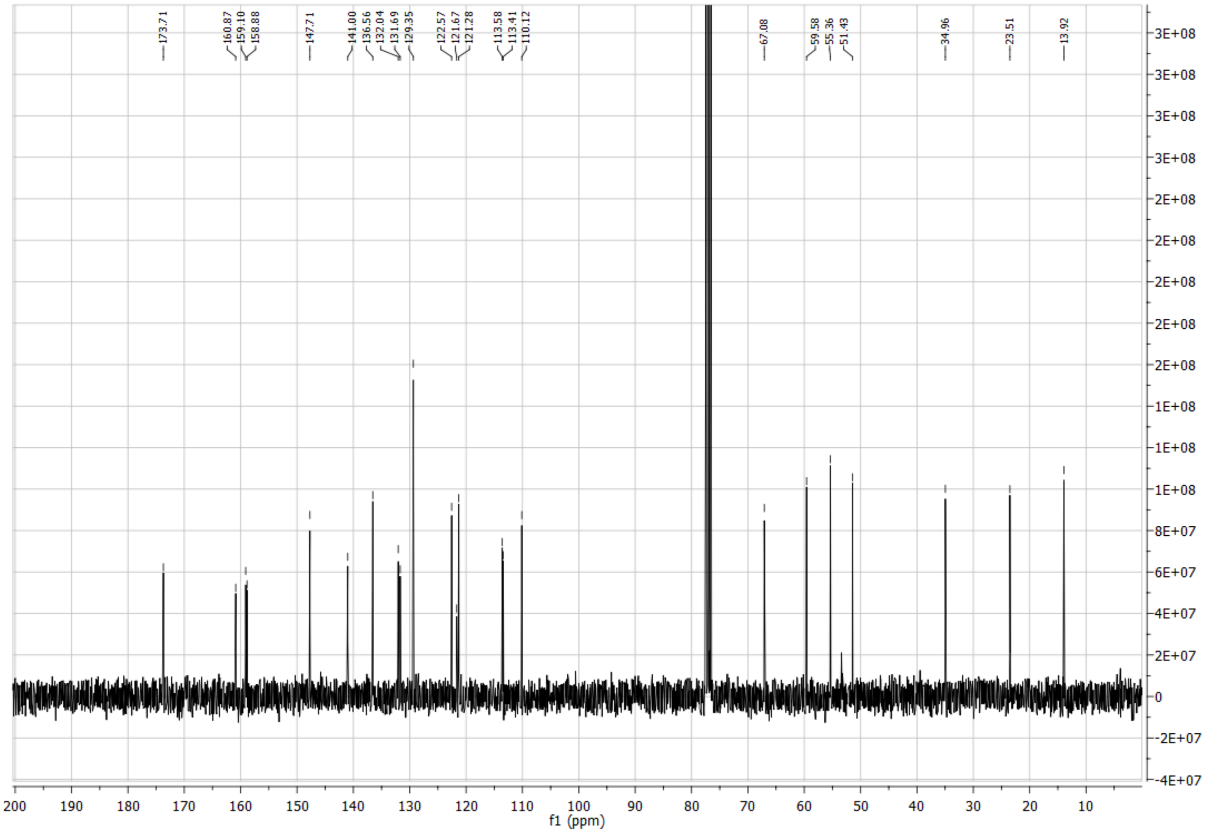
173.71
160.87
159.10
158.88
147.71
141.00
136.56
132.04
131.69
129.35
122.57
121.67
121.28
113.58
113.41
110.12
67.08
59.58
55.36
51.43
34.96
23.51
13.92
f1 (ppm)

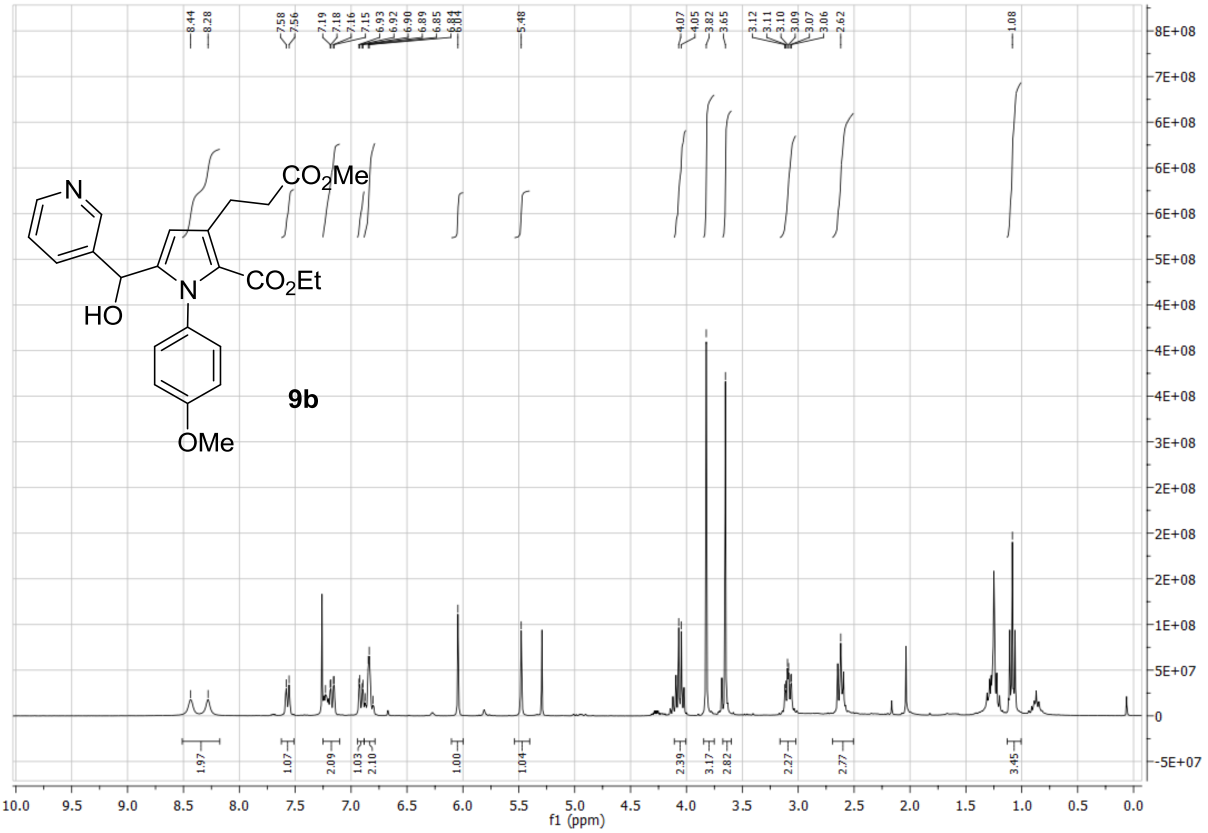
CO2Me
N
CO2Et
HO
N
OMe
9b
8.44
8.28
7.58
7.56
7.19
7.18
7.16
7.15
6.93
6.92
6.90
6.89
6.85
6.84
6.04
5.48
4.07
4.05
3.82
3.65
3.12
3.11
3.10
3.09
3.07
3.06
2.62
1.08
1.97
1.07
2.09
1.03
2.10
1.00
1.04
2.39
3.17
2.82
2.27
2.77
3.45
f1 (ppm)

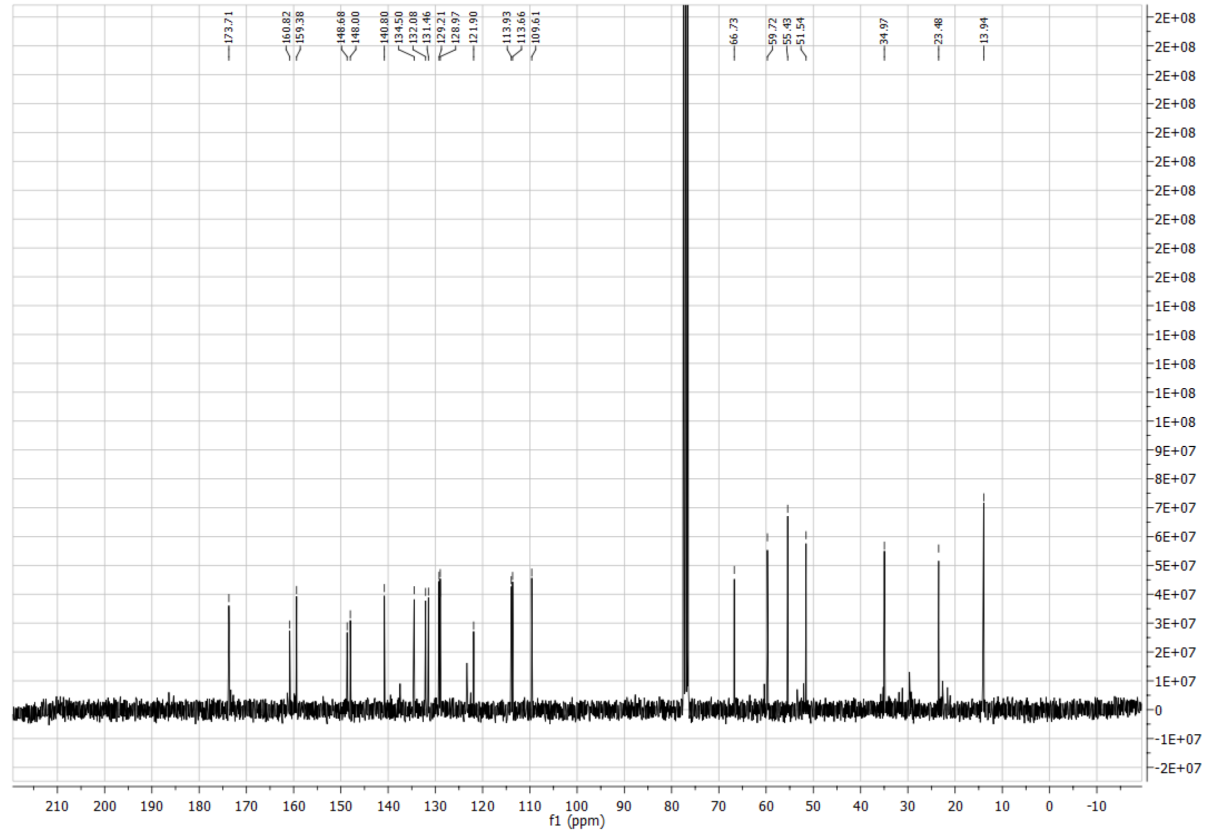
173.71
160.82
159.38
148.68
148.00
140.80
134.50
132.08
131.46
129.21
128.97
121.90
113.93
113.66
109.61
66.73
59.72
55.43
51.54
34.97
23.48
13.94
f1 (ppm)

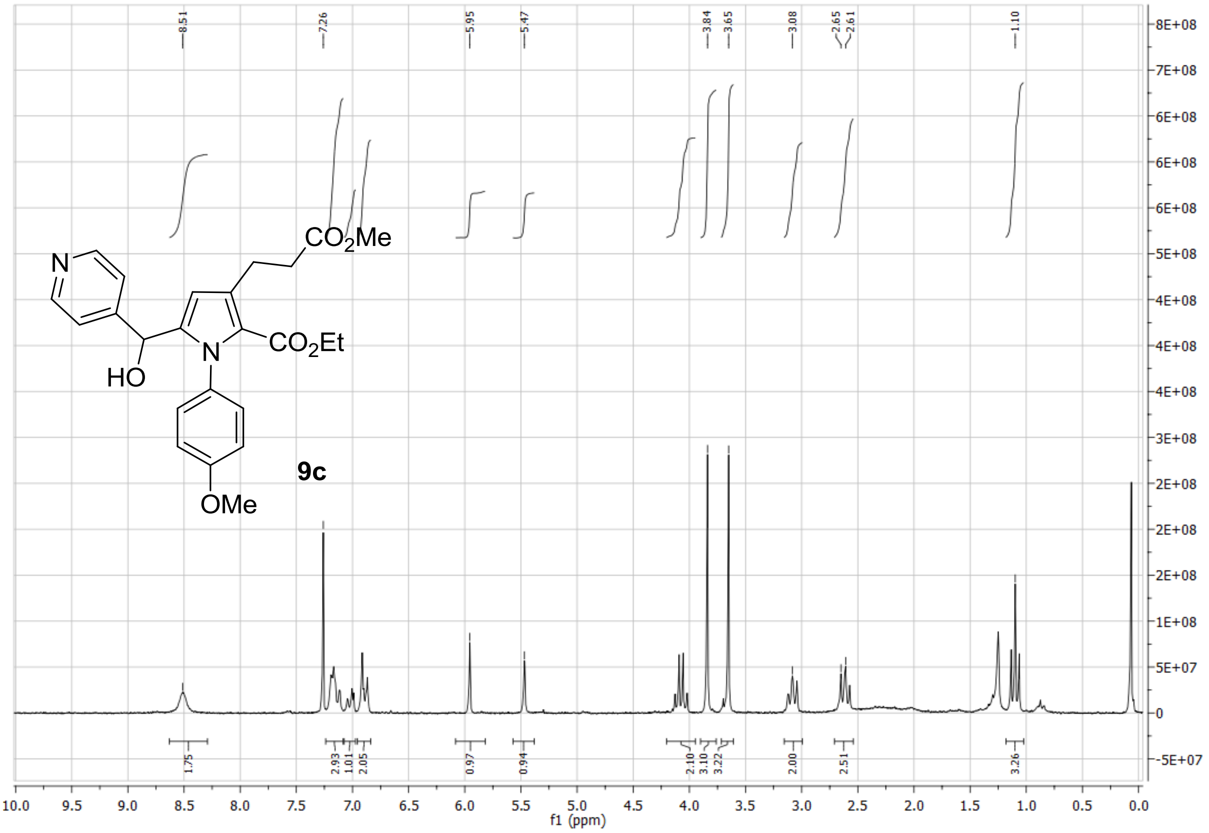

8.51
7.26
5.95
5.47
3.84
3.65
3.08
2.65
2.61
1.10
1.75
2.93
1.01
2.05
0.97
0.94
2.10
3.10
3.22
2.00
2.51
3.26
CO2Me
N
CO2Et
HO
N
OMe
9c
f1 (ppm)

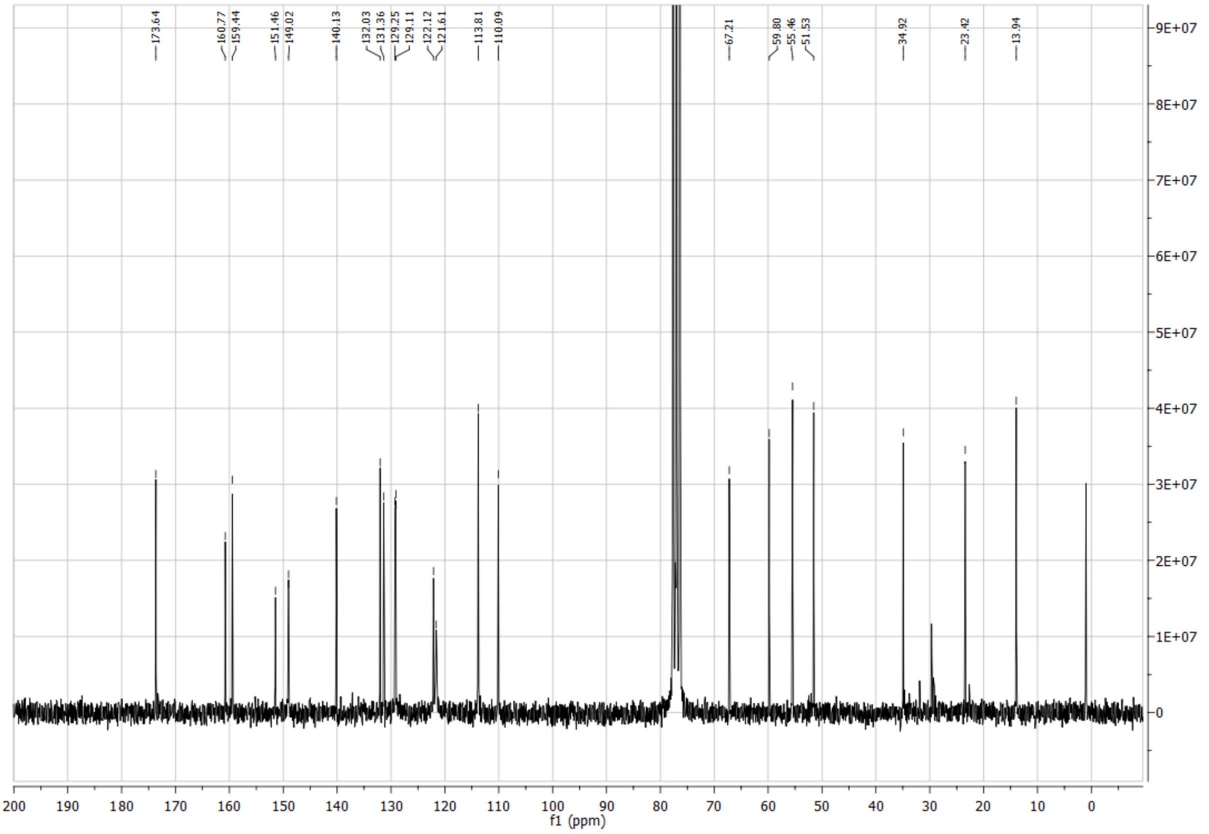

173.64
160.77
159.44
151.46
149.02
140.13
132.03
131.36
129.25
129.11
122.12
121.61
113.81
110.09
67.21
59.80
55.46
51.53
34.92
23.42
13.94
f1 (ppm)

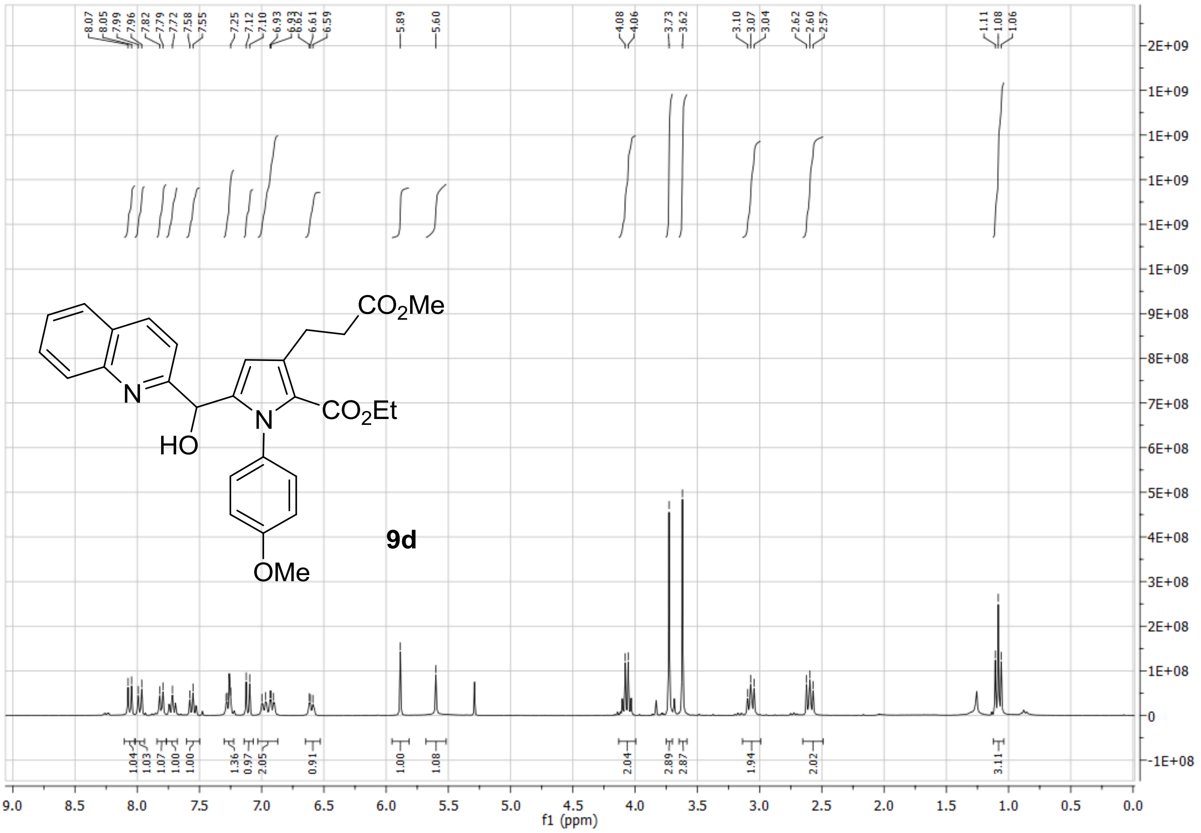
8.07 8.05 7.99 7.96 7.82 7.79 7.72 7.58 7.55 7.25 7.12 7.10 6.93 6.93 6.92 6.61 6.59 5.89 5.60 4.08 4.06 3.73 3.62 3.10 3.07 3.04 2.62 2.60 2.57 1.11 1.08 1.06
CO2Me
N
HO
N
CO2Et
OMe
9d
1.04 1.03 1.07 1.00 1.00 1.36 0.97 2.05 0.91 1.00 1.08 2.04 2.89 2.87 1.94 2.02 3.11
f1 (ppm)

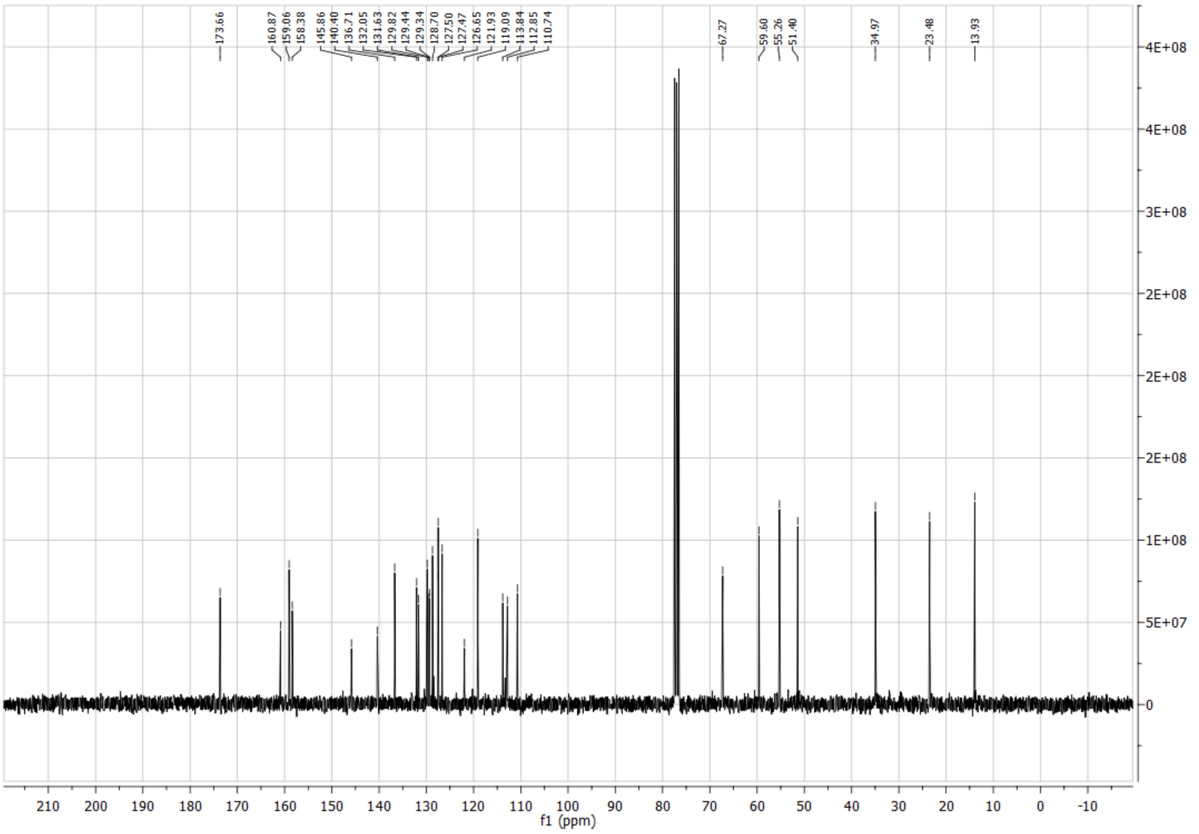
173.66 160.87 159.06 158.38 145.86 140.40 136.71 132.05 131.63 129.82 129.44 129.34 128.70 127.50 127.47 126.65 121.93 119.09 113.84 112.85 110.74 67.27 59.60 55.26 51.40 34.97 23.48 13.93
f1 (ppm)

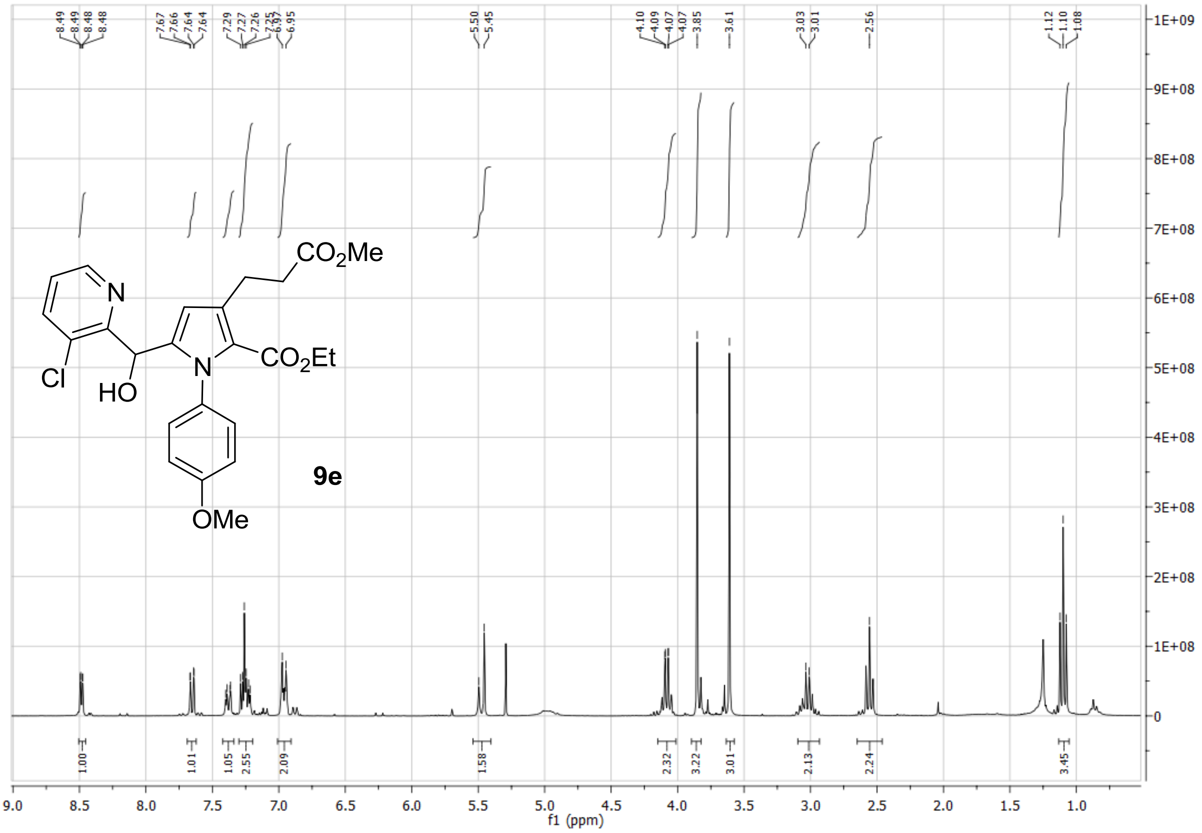
CO2Me
N
Cl
HO
N
CO2Et
OMe
9e
f1 (ppm)

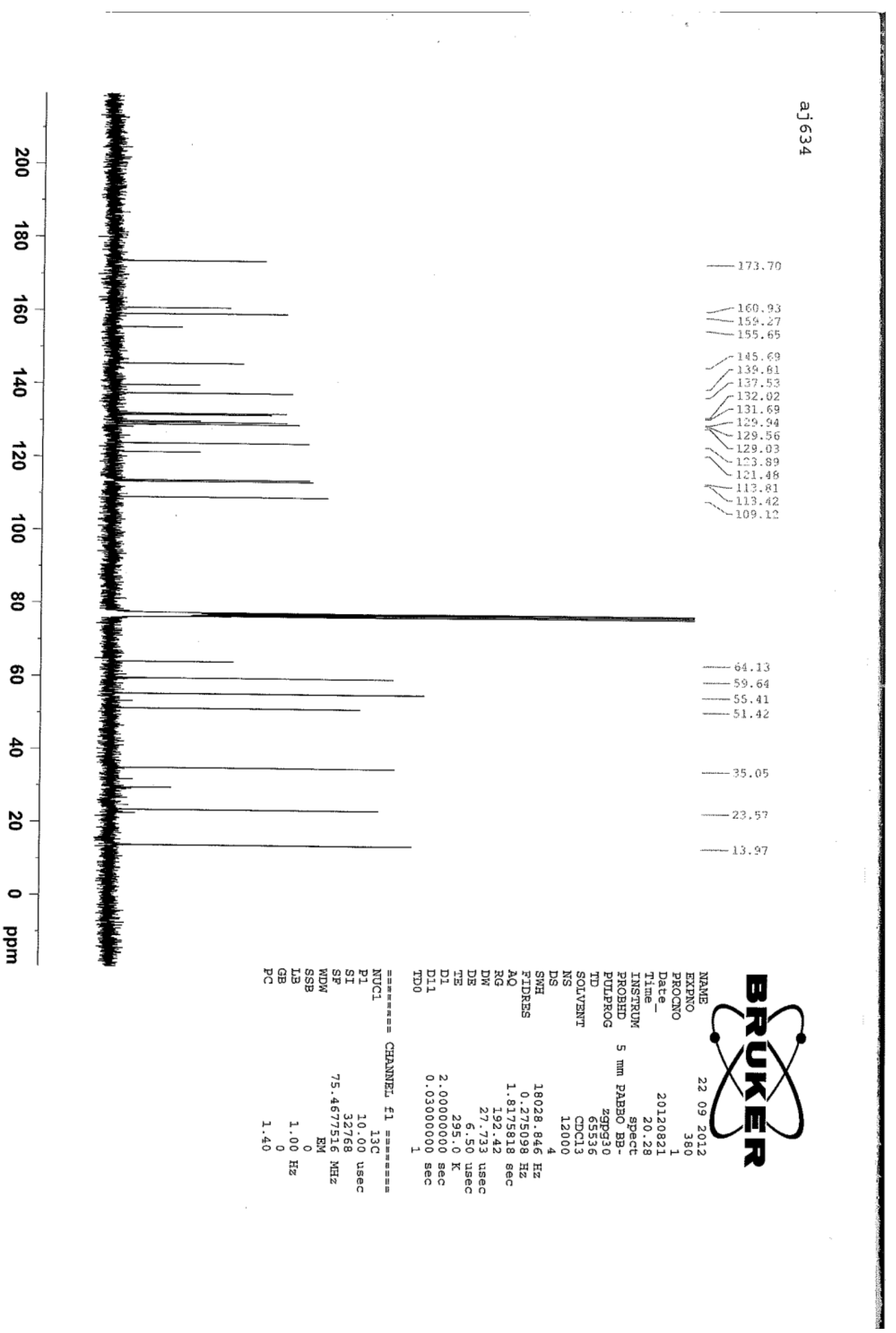
aj634
173.70
160.93
159.27
155.65
145.69
139.81
137.53
132.02
131.69
129.94
129.56
129.03
123.89
121.48
113.81
113.42
109.12
64.13
59.64
55.41
51.42
35.05
23.57
13.97
200
180
160
140
120
100
80
60
40
20
0
ppm
BRUKER
NAME 22 09 2012
EXPNO 380
PROCNO 1
Date_ 20120821
Time 20.28
INSTRUM spect
PROBHD 5 mm PABBO BB-
PULPROG zgpg30
TD 65536
SOLVENT CDCl3
NS 12000
DS 4
SWH 18028.846 Hz
FIDRES 0.275098 Hz
AQ 1.8175818 sec
RG 192.42
DW 27.733 usec
DE 6.50 usec
TE 295.0 K
D1 2.00000000 sec
D11 0.03000000 sec
TD0 1
======== CHANNEL f1 ========
NUC1 13C
P1 10.00 usec
SI 32768
SF 75.4677516 MHz
WDW EM
SSB 0
LB 1.00 Hz
GB 0
PC 1.40

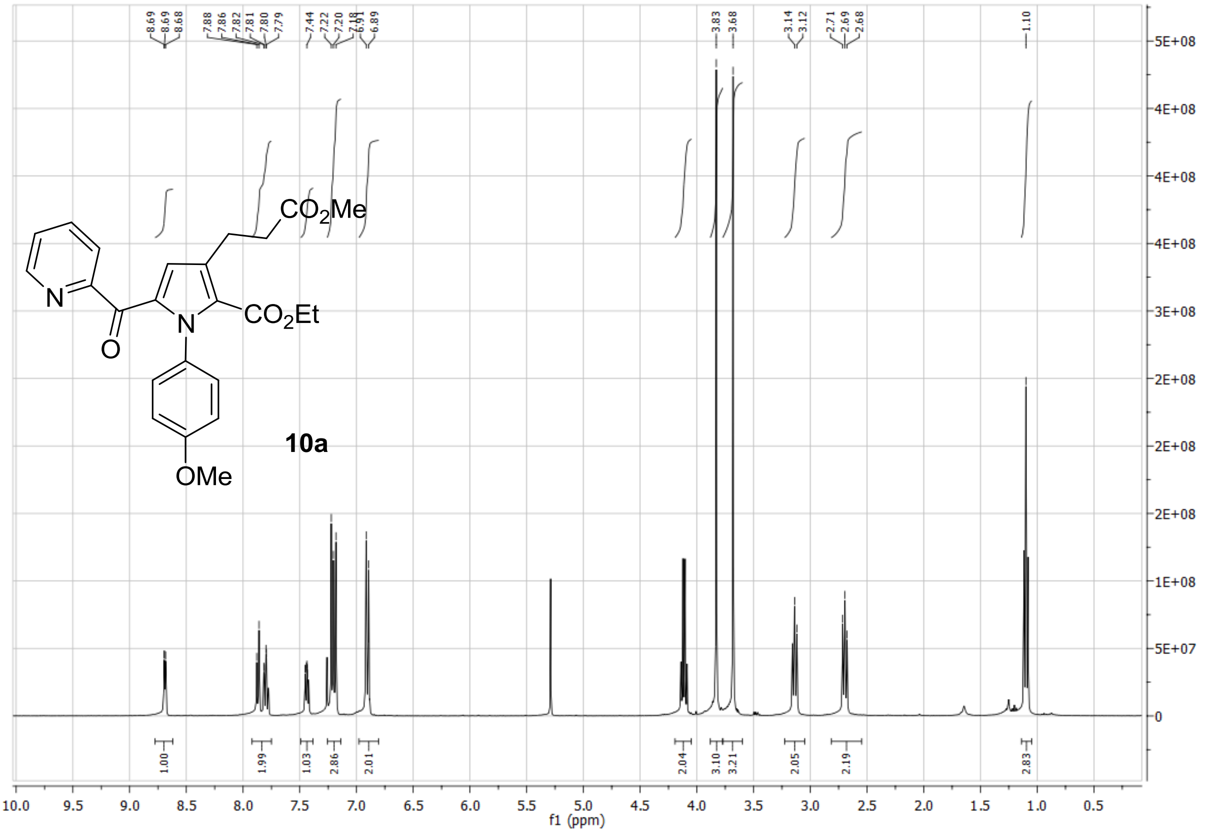
CO2Me
N
O
N
CO2Et
OMe
10a
f1 (ppm)

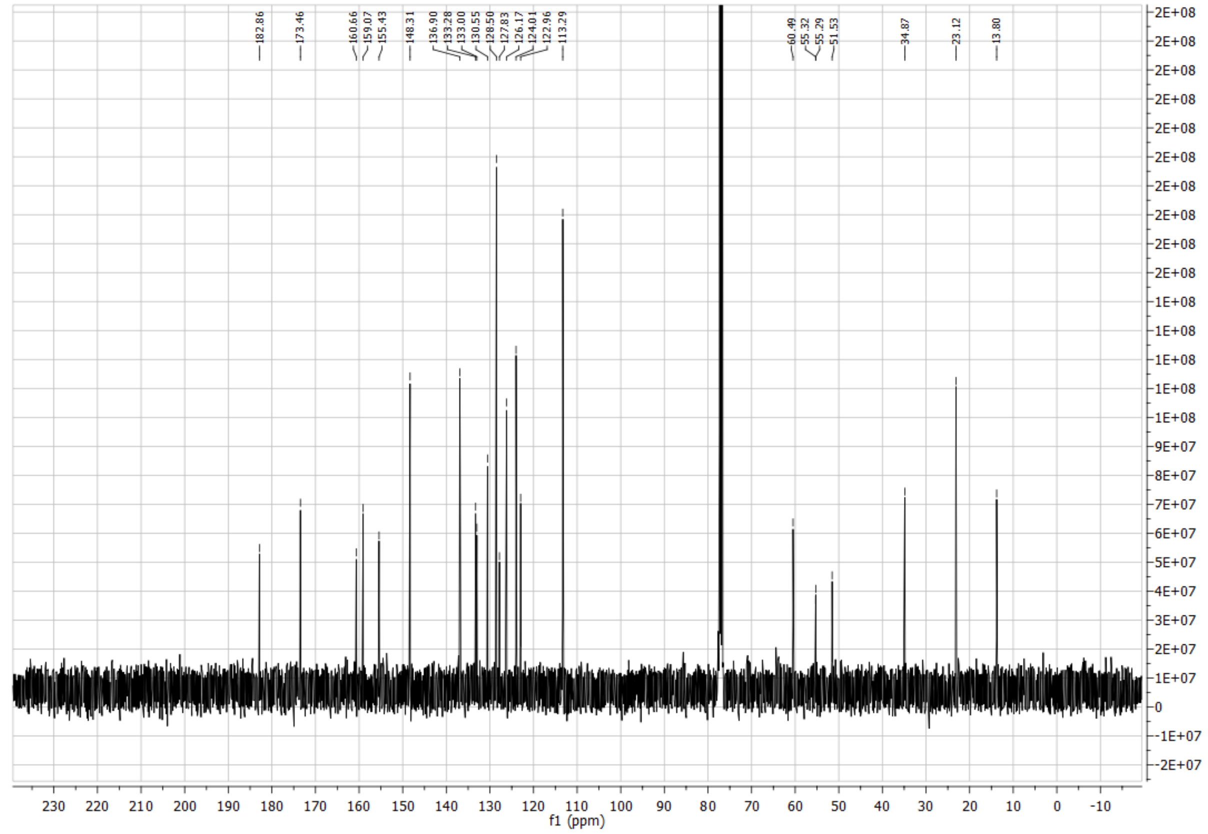
f1 (ppm)
